# Gene Expression Patterns in Larval *Schistosoma mansoni* Associated with Infection of the Mammalian Host

**DOI:** 10.1371/journal.pntd.0001274

**Published:** 2011-08-30

**Authors:** Sophia J. Parker-Manuel, Alasdair C. Ivens, Gary P. Dillon, R. Alan Wilson

**Affiliations:** 1 University of York, Heslington, York, United Kingdom; 2 Fios Genomics Ltd. ETTC, Edinburgh, United Kingdom; McGill University, Canada

## Abstract

**Background:**

The infective schistosome cercaria develops within the intramolluscan daughter sporocyst from an undifferentiated germ ball, during which synthesis of proteins essential for infection occurs. When the aquatic cercaria locates the mammalian host it rapidly penetrates into the epidermis using glandular secretions. It then undergoes metamorphosis into the schistosomulum, including replacement of its tegument surface membranes, a process taking several days before it exits the skin. Patterns of gene expression underlying this transition have been characterised.

**Methods and Principal Findings:**

All gene models from the *S. mansoni* genome (www.GeneDB.org) were incorporated into a high-density oligonucleotide array. Double-stranded cDNA from germ balls, cercariae, and day 3 schistosomula was hybridised to the array without amplification. Statistical analysis was performed using Bioconductor to reveal differentially transcribed loci. Genes were categorised on the basis of biological process, tissue association or molecular function to aid understanding of the complex processes occurring. Genes necessary for DNA replication were enriched only in the germ ball, while those involved in translation were up-regulated in the germ ball and/or day 3 schistosomulum. Different sets of developmental genes were up-regulated at each stage. A large number of genes encoding elastases and invadolysins, and some venom allergen-like proteins were up-regulated in the germ ball, those encoding cysteine and aspartic proteases in the cercaria and schistosomulum. Micro exon genes encoding variant secreted proteins were highly up-regulated in the schistosomulum along with tegument and gut-associated genes, coincident with remodelling of the parasite body. Genes encoding membrane proteins were prominently up-regulated in the cercaria and/or day 3 schistosomulum.

**Conclusions/Significance:**

Our study highlights an expanded number of transcripts encoding proteins potentially involved in skin invasion. It illuminates the process of metamorphosis into the schistosomulum and highlights the very early activation of gut-associated genes whilst revealing little change in the parasite's energy metabolism or stress responses.

## Author Summary

The schistosome cercaria develops from undifferentiated germ balls within the daughter sporocyst located in the hepatopancreas of its snail intermediate host. This is where the proteins it uses to infect humans are synthesised. After a brief free life in fresh water, if the cercaria locates a host, it infects by direct penetration through the skin. It then transforms into the schistosomulum stage, adapted for life in human tissues. We have designed a large scale array comprising probes representing all known schistosome genes and used it in hybridisation experiments to establish which genes are turned on or off in the parasite during these stages in its life cycle. Genes encoding proteins involved in cell division were prominent in the germ ball along with those for proteases and potential immunomodulators, deployed during skin penetration. The non-feeding cercaria was the least active at synthesising proteins. Conversion to the schistosomulum was accompanied by transcription of genes involved in body remodeling, including production of a new outer surface, and gut activation long before ingestion of red blood cells begins. Our data help us to understand better the proteins deployed to achieve infection, and subsequent adaptations necessary for establishment of the parasite in the human host.

## Introduction

Schistosomiasis mansoni remains an important water-borne disease of humans in Sub-Saharan Africa and parts of South America. Transmission between humans and the aquatic intermediate molluscan host is effected by a miracidium. This free-swimming larva hatches from eggs excreted in the faeces and penetrates into the snail. There follows a phase of asexual multiplication within the snail, before the non-feeding infective cercariae emerge. These have a free life of only hours during which they must locate a human host or perish. Infection occurs when a cercaria penetrates through the skin and transforms into the schistosomulum stage. There is then a period of physiological and morphological adaptation to the new environment, lasting several days, before the parasite locates a blood or lymphatic vessel to exit the skin and begin its intravascular migration to the portal system. The biological processes associated with the transition from snail haemolymph via fresh water to mammalian tissues are the key to an understanding of the infection process. Indeed, it could be argued that preventing parasite establishment would provide the optimum control strategy for the disease. The secretions used by the cercaria to enter the skin have been a focus of interest for decades, latterly using biochemical [1] and proteomic techniques [2], [3]. These studies have revealed the importance of serine- and metallo-proteases plus potential immunomodulators released by the parasite to gain entry and establish in the skin. However, the relative paucity of parasite material coupled with the limited sensitivity of proteomic techniques means that we have only a partial picture of one small aspect of the transition from snail to human. For example, virtually nothing is known about processes associated with development of the germ balls that mature into cercariae within the daughter sporocyst, nor is the transformation into the schistosomulum well characterised. The cercarial tegument is known to be shed and replaced by the novel double bilayer structure [4] accompanied by the appearance of glucose transporters on the schistosomulum surface, doubtless to facilitate nutrient uptake [5]. The switch from aerobic to anaerobic metabolism has also been noted [6]. The highly sensitive methods now available to characterise gene expression present an opportunity to gain a deeper understanding of this important transition in the life cycle.

Microarrays have become a widely used tool for comparing transcription levels between different biological samples; it is good practice to use at least three biological replicates (i.e. material from separate organisms) to get statistically significant results for each comparison made. A variety of schistosome arrays has previously been designed and used to answer distinct questions about the parasite's biology. These have included differences in gene transcription between female and male adult worms [7], [8], with laser-capture micro-dissection added to facilitate comparison of gastrodermis and vitellaria [9], and changes in transcription between different life cycle stages [10]–[13]. Germane to our current study, a custom cDNA array, comprising 6000 features from the lung stage larva was used to identify transcripts enriched at the lung stage compared to six other life cycle stages [12]. The same array was also used to characterise differences in transcription pattern between schistosomula transformed from normal and radiation-attenuated cercariae, and cultured *in vitro* for four, seven and ten days [14].

Other arrays, comprising 12–38,000 synthetic oligonucleotide probes have been used to investigate a range of life cycle stages. These arrays were based on ESTs and contigs available pre-2006 at the DCFI *S. mansoni* Gene Index (formerly the TIGR Gene Index) [11], [15] or ESTs and contigs available at GenBank and the Wellcome Trust Sanger Institute ftp site in May 2005 [13]. Infected snail hepatopancreas, cercariae and adult worms, have been compared using such oligonucleotide arrays, with uninfected snail tissue as a control for the first of these samples [11]. It was reported that the intramolluscan parasite has high levels of transcripts encoding proteins involved in translation and quality control, cell death and ubiquitination. In the cercaria, highly expressed genes were mainly involved in mitochondrial function, enabling the energy production necessary for swimming. However, it was noted that the cercaria was less transcriptionally active than the other stages studied. Transcription levels at 15 distinct points throughout the parasite life cycle have also been compared [13]. Data analysis focussed on three gene families, fucosyl transferases, tetraspanins, and G protein-coupled receptors (GPCRs) proffered as potential intervention targets. Finally transcription in 3 hr and 5 day schistosomula, cultured *in vitro* +/− erythrocytes, was compared with cercariae as the baseline [15]. The most apparent changes were the up-regulation of genes involved in blood feeding, tegument and cytoskeleton development, cell adhesion and stress. Although the annotated *S. mansoni* genome with standardised nomenclature for predicted gene models was published in 2009 [16], the above microarray studies have used other nomenclatures and annotations for their constituent ESTs and contigs. This makes specific inter-study comparisons about changes in the transcription of named genes extremely cumbersome and when attempted, points up considerable discrepancies in annotation.

We report here the design and use of the most comprehensive microarray platform for *S. mansoni* to gain insights into infection of the mammalian host. The array was used to probe transcripts from three life cycle stages, intramolluscan germ balls, free-living cercariae, and ‘skin’ schistosomula. We use the term ‘germ ball’ to encompass all stages of embryonic development up to, but excluding, the mature cercaria that comprises approximately 1000 cells [17], differentiated into tissues and organs. The germ balls were essential as it has been shown that many proteins used by the cercaria for host entry are transcribed and translated during its development in the snail [2], [18]. They were obtained by microdissection of snails 22–26 days post-infection. Day 3 schistsosomula were chosen because by that point, metamorphosis from the cercaria is nearing completion, they have adapted to life in mammalian tissues and are ready to begin intravascular migration. Such schistosomula, transformed and cultured by the methods we used, are able to mature if transferred into the murine host [19]. They are biologically comparable to *ex vivo* worms and can be produced in large quantities. Sufficient RNA was obtained from all three life cycle stages for hybridisation to the array without PCR amplification. A comprehensive analysis of greater than two-fold changes in transcription between the life cycle stages is presented.

## Methods

### Ethics statement

The procedures involving animals were carried out in accordance with the UK Animals (Scientific Procedures) Act 1986, as authorised on personal and project licences issued by the UK Home Office. The study protocol was approved by the Biology Department Ethical Review Committee at the University of York.

### Biological material

All parasite material was from a Puerto Rican isolate of *S. mansoni* maintained at the University of York by passage through NMRI strain mice and albino *Biomphalaria glabrata* snails. Developing germ balls from daughter sporocysts were obtained from snails infected with 40 miracidia each and dissected carefully in filter-sterilised 50% PBS (pH 7.4.) 22–26 days later, before cercarial maturity. Obvious snail material was removed and freed germ balls at all stages of development were accumulated on ice until use. Cercariae were collected from snails infected with 10 miracidia each. Five weeks after infection the snails were placed in the dark for two days and then illuminated in approximately 10 mls aerated tap water for two hours to induce shedding. The emerging cercariae were gravity-concentrated by cooling on ice for one hour, which prevented swimming. Skin stage schistosomula were obtained by mechanical transformation of cercariae and separation of their bodies which were cultured for three days *in vitro* as previously described [19]. They were then recovered and washed twice in RPMI before processing [19].

### RNA extraction, quantification and quality assessment

RNA was extracted from the three larval stages by homogenisation in TRIzol (Invitrogen, Paisley, UK) at approximately 1 ml per 100 µl tissue. The RNA was extracted as per the manufacturer's instructions, with the addition of DEPC-treated high salt solution (0.8 M sodium citrate and 1.2 M NaCl) at the isopropanol step, to remove glycoprotein. RNA was isopropanol-precipitated overnight at −80°C with 1 µl Glycoblue (15 mg/ml; Ambion) to aid the process, and visualisation of the pellet. It was recovered by centrifugation at 12,000× g for 30 minutes at 4°C. The pellets were washed with 70% ethanol, and allowed to air-dry at room temperature before being resuspended in 300 µl DEPC-treated water. RNA was quantified using a Nanodrop ND-1000 Spectophotometer (Nanodrop Products Fisher, Wilmington, Delawere, USA) and quality assessed using a 2100 Bioanalyzer PicoChip (Agilent, Wokingham, UK).

### Design of the microarray

The predicted genes from version D of the *S. mansoni* genome assembly as of June 2008 (www.GeneDB.org) formed the input for the array design along with all *S. mansoni* ESTs available at GeneDB.org whose direction was known, compiled using phrap (http://www.phrap.org/phredphrapconsed.html). The input data were broken up into sequential 50mers offset by one base each time, and redundant sequences were removed using FAlite.pm (Ian Korf; http://homepage.mac.com/iankorf/) and associated Perl scripts. The unique sequences were mapped back to the genome assembly using ‘exonerate’ (http://www.ebi.ac.uk/~guy/exonerate/). From a map-ordered list, every 13^th^ 50mer was chosen as a probe. No selection was made for the number of probes per predicted transcript. The design was sent to Roche-NimbleGen, who made some minor refinements for ease of synthesis and constructed the arrays using digital micromirror technology [20]. There were 385K features on the array comprising 377,598 *S. mansoni* sequences and 11,613 random sequences for hybridisation controls.

### cDNA synthesis, experimental design and hybridisation

Double stranded cDNA for hybridisation was synthesised from total RNA using SuperScript Double-Stranded cDNA synthesis kits (Invitrogen) according to the protocol supplied by Roche-NimbleGen. The resulting cDNA was pooled such that separate biological replicates were obtained i.e. no parasite homogenates were split across replicates. Roche-NimbleGen were supplied with at least 2.7 µg of double stranded cDNA for three biological replicates each from germ balls, cercariae, and day 3 schistosomula to perform the hybridisations. Each biological replicate of cDNA was labelled with Cy3 and hybridised to the array for 16 to 20 hours at 42°C. Slides were washed, and dried before fluorescence data were read using a Roche-NimbleGen MS 2000 Scanner with NimbleScan software.

### Data analysis

Roche-NimbleGen supplied background-corrected data. All subsequent statistical analysis was carried out using programmes from the Bioconductor suite [21]. The data were quality-assessed by visual inspection of graphical representations of the raw probe level data. Box plots were drawn using the boxplot function from the graphics package. Correlation data were calculated using the cor function from the stats package and heatmaps were made by calling the heatmap.2 function in the gplots package. All arrays passed the quality assessment. Next, the data were quantile-normalised using the normalizeBetweenArrays function in the limma package. This ensures identical distributions of the data [22]. Following normalisation, the probe level data were summarised to yield ‘gene level’ data. The probes were re-mapped to the *S. mansoni* gene predictions at www.geneDB.org (version F) using ‘exonerate’. If a probe matched an *S. mansoni* predicted gene (Smp) locus with an e value <1^−05^ by both nucleotide and protein BLAST, the probe was annotated to that Smp locus. The intensity value for each locus was the mean intensity of all the probes by which it was represented. The resulting gene level data were the input for the differential expression analysis, which was carried out using the limma package. First a linear model fit was performed. This reduced the data for each gene to a mean value from each of the life cycle stages. Next, differential expression data were obtained by performing a contrast analysis. This compares the transcription level of each gene in the following contrasts:

germ ball : cercaria
germ ball : day 3 schistosomulum
day 3 schistosomulum : cercaria

Multiple testing was corrected for using the eBayes function which employs the method of Benjamini and Hochberg [23]. This gives an adjusted P value (adjP). Genes which were differentially expressed above a two fold cut-off between any two stages with an B>3 were chosen for further analysis. The B value is the log (odds that a gene is differentially expressed). For example if B = 3, the odds that a gene is expressed is e^3^ = 20, or 1 in 20, corresponding to a probability of 95%. Log2 quantile-normalised probe level data from the array are deposited at the public database Gene Expression Omnibus (http://www.ncbi.nlm.nih.gov/geo) under accession numbers GSE22037 and GPL10466. For ease of visualisation, statistically significant expression values were converted to relative fold change, with the stage having the lowest expression for each gene set to unity. This facilitates analysis of changes in gene expression associated with the infection process. The actual baseline expression values may be different for each gene, and comparisons of absolute expression levels between genes, based on the charts and tables shown here, are not valid. However, the patterns of gene expression may be compared between genes. Where a data bar is missing for a particular life cycle stage, this is because either no statistically significant gene expression was observed for that life cycle stage, or there was a less than two-fold difference. Finally a hypergeometric test was carried out using the Category package to discover whether particular GO terms were over-represented in the differentially expressed genes compared to the ‘gene universe’ of Smp gene models on the array.

As an aid to understanding biological processes associated with the transition of the parasite from the snail, via fresh water to the mammalian host tissues, we grouped the *S. mansoni* genes into useful categories. Some of these (DNA replication, translation, energy metabolism, lipid metabolism, and membrane) were based purely on the classification of genes by Gene Ontology (http://www.geneontology.org/). In creating a muscle tissue category, we used the GO term ‘actin binding’. Two of the tissue categories, ‘tegument’ and ‘alimentary tract’ were created using our proteomic analyses [24]–[28]. In the case of ‘alimentary tract’, as four proteins containing saposin domains had already been identified in worm vomitus, we searched the genome database for other genes encoding saposins and added them to this category. The custom category ‘defence against stress’ was compiled from the literature on schistosomes [29]–[31]. The VAL and MEG categories were based on recently published compilations [32], [33]. Genes encoding proteins involved in lipid synthesis were culled from supplementary table 11 of the genome paper [16]. The lists of proteases classified by catalytic type (http://merops.sanger.ac.uk/) were abstracted from the *S. mansoni* genome supplementary table 18 [16]. The ‘development’ category comprised the neural development and TGFβ signalling genes highlighted in the genome paper (supplementary tables 9 and 15, respectively [16]) along with those genes annotated to the GO term ‘Wnt receptor signaling pathway’ while the protein glycosylation category was created by assiduous interrogation of the *S. mansoni* genome database.

### qPCR

RNA from each of the biological replicates was reserved for qPCR to validate the microarray results. Total RNA (0.413 µg) from each sample was reverse-transcribed using Superscript II (Invitrogen) and primed with oligo-dT according to the manufacturer's instructions, in a reaction volume of 20 µl. After the reaction, the volume was made up to 100 µl with DEPC-treated water. Relative quantitation was carried out using SYBR green PCR Master Mix (Applied Biosystems) with 1 µl of cDNA per 25 µl reaction. The PCR was carried out using an ABI7300 (Applied Biosystems) according to the manufacturer's instructions with 18S ribosomal RNA [14] as the endogenous control, run for each sample on each plate. Genes chosen and their primer pairs are detailed in Table S1, with primers designed using PrimerExpress (Applied Biosystems). There were three technical replicates per biological replicate for each gene. Relative quantification was calculated by the ΔΔCT method using Applied Biosystems Sequence Detection Software version 1.2.3 7000 system.

## Results and Discussion

The availability of the genome assembly and gene models along with ESTs has permitted the design and construction of the most comprehensive array to date for this medically important parasite. With this we have investigated the pattern of differential gene transcription before, during and after infection of the mammalian host. In effect we are studying a linear biological process in which a single diploid germ cell divides to produce a germ ball within the body of the intramolluscan daughter sporocyst. This in turn, differentiates into the cercaria that transforms into the schistosomulum, that will ultimately become the adult worm. Note that genes encoding proteins for schistosomulum migration within the mammalian host are first transcribed in the cercaria, but there is little embryogenesis involved in transformation, which is better described as a metamorphosis involving redifferentiation of existing cells and tissues.

The levels of gene transcription sampled by the array were independently validated on the same biological replicates using qPCR. For the selected genes, in each of the life cycle comparisons, the log_2_ fold change was determined. All qPCR log_2_ fold changes were plotted against those microarray data with a P≤0.05 (n = 15) as the independent variable (Figure S1). A linear regression produced a slope of 0.5 and an r^2^ value of 0.85 (P<0.0001). Treating the datasets as normally distributed independent variables, a Pearson correlation coefficient was calculated giving an *r* value of 0.89 (P<0.0001). We can therefore have confidence in the predictions of the array. Large numbers of loci were differentially regulated above the two-fold threshold: 1731 between germ ball and cercaria, 1431 between germ ball and day 3 schistosomulum, and 1066 between cercaria and day 3 schistosomulum.

The singularity of the germ ball, relative to the other two stages is explicable both in terms of the developmental processes that take place, and the environments encountered. Relatively few genes were transcribed uniquely or even enriched in the cercaria. This is unsurprising given that the non-feeding cercaria must rely on endogenous reserves of metabolites for synthetic processes. In this respect we can make a distinction between cercarial proteins manufactured for a single event (gland cell contents synthesised in the germ ball) and those needed continuously to maintain viability and activity (swimming). As an aid to understanding, the differentially transcribed genes are considered below in the sequence: biological process, development and tissues, and molecular function, each with appropriate subdivisions.

### Biological process

This term encompasses the basic functions that might be expected to occur in the ‘average’ schistosome cell.

#### DNA replication and cell division

Although only 5% of the *S. mansoni* genes in the GO category ‘DNA replication’ were differentially regulated, all of them were enriched in the germ ball, reflecting the cell division occurring there but not in the cercaria or schistosomulum (Table 1). The highest change (22 fold) was exhibited by the mitotic cyclin B, followed by histone H2A (18 fold). Nine minichromosome maintenance genes (MCMs) were prominent, including the six that encode the hexamer ring helicase forming part of the pre-replication complex. CDC45 which is rate-limiting for DNA replication licensing in human cells [34], GINS, (also part of the ‘unwindosome’) and the inhibitory Geminin were up-regulated. Also noteworthy were seven DNA polymerase subunits, four DNA repair enzymes, three histones, three cyclins which control cell cycle progression, a TGF beta receptor, DNA primase, DNA ligase, proliferating cell nuclear antigen (PCNA), and several replication factors. The exception, the DNA repair helicase, rad 25, was up-regulated 2-fold in the day 3 schistosomulum compared to the cercaria. Only one RNA polymerase was differentially transcribed, suggesting that each of the stages performs transcription at a similar rate.

**Table 1:** DNA replication and cell division.

Annotation	Gene ID	GB	C	D3
cyclin B	Smp_082490	22.63	1.00	1.89
histone H2A	Smp_086860	18.09	1.00	2.25
MCM4	Smp_172530	14.57	1.00	1.62
Geminin	Smp_176260	13.00	1.06	1.00
MCM2	Smp_054840	11.11	1.00	1.66
cyclin d	Smp_153660	10.78	1.00	1.21
MCM5	Smp_143490	9.42	1.00	1.38
MCM6	Smp_094140	9.04	1.00	1.92
DNA primase large subunit	Smp_079050.1	8.24	1.08	1.00
MCM3	Smp_037590	7.84	1.00	1.97
MCM2	Smp_079560	7.15	1.00	1.04
TGF-beta receptor type I	Smp_067260	7.00	1.03	1.00
histone H2A	Smp_002930	6.63	-	1.00
CDC 45-related	Smp_047610	6.27	1.01	1.00
MCM7	Smp_032500	6.26	1.52	1.00
DNA photolyase	Smp_033700	6.03	1.00	1.52
MCM3	Smp_113350	6.00	1.00	1.40
RecQ protein 4	Smp_069600	5.62	1.00	1.29
replication factor C	Smp_096360	5.19	1.00	-
DNA polymerase epsilon subunit B	Smp_124120	5.12	1.00	1.57
Proliferating cell nuclear antigen	Smp_046500.1	4.98	1.00	2.22
cyclin B3	Smp_047620	4.92	2.04	1.00
DNA polymerase alpha catalytic subunit	Smp_178260	4.90	1.01	1.00
GINS	Smp_100380	4.85	1.00	-
rad1 DNA damage checkpoint protein	Smp_075110	4.62	1.34	1.00
DNA repair protein RAD51	Smp_124230	4.61	1.34	1.00
centromere protein A	Smp_040020	4.59	1.00	1.33
flap endonuclease-1	Smp_186980	4.39	1.00	2.15
histone H2A	Smp_035980	4.29	1.50	1.00
DNA primase	Smp_172020	3.84	1.00	1.00
Proliferating cell nuclear antigen	Smp_046500.2	3.82	1.00	-
nuclease	Smp_106040	3.69	1.00	-
MCM1	Smp_151560	3.65	1.00	1.02
replication factor C	Smp_066260	3.48	1.00	-
exonuclease	Smp_012480	2.88	1.00	1.48
MutS	Smp_169120	2.85	1.00	1.24
Telomere binding protein	Smp_076050	2.84	1.00	-
structural maintenance of chromosomes smc2	Smp_171710	2.65	1.00	1.36
DNA polymerase delta small subunit	Smp_076660	2.65	1.00	-
DNA polymerase I	Smp_055500	2.64	1.03	1.00
ribonucleoside-diphosphate reductase	Smp_009030	2.59	1.00	-
replication factor A 1, rfa1	Smp_121800	2.39	1.00	1.33
DNA polymerase delta catalytic subunit	Smp_087010	2.28	1.00	-
DNA polymerase gamma	Smp_161380	2.20	1.00	1.79
DNA ligase I	Smp_019840.2	2.16	1.00	1.13
DNA polymerase eta	Smp_136180	2.04	1.00	-
ap endonuclease	Smp_051320	2.01	1.00	-
rad25/xp-B DNA repair helicase	Smp_165580	1.81	1.00	2.07

#### Translation

Only 31 of the 780 genes annotated to the GO term ‘translation’ were differentially regulated in the three comparisons. None of the changes were exceptional, the highest being 4.95 fold (Table 2). The vast majority (27; ten ribosomal proteins, ten t-RNA synthetases, five initiation factors and two translation elongation factors) were most highly transcribed in the germ ball, but 8/31 were also up-regulated in the day 3 schistosomulum; only three had the highest level of transcription in the cercaria. These data indicate that the germ ball is the most translationally active, the cercaria the least.

**Table 2:** Expression patterns of genes encoding proteins involved in translation.

Annotation	ID	GB	C	D3
eukaryotic translation initiation factor 2c	Smp_179320	4.59	1.60	1.00
eukaryotic elongation factor 2 kinase	Smp_072980	3.93	1.00	2.30
lysyl-tRNA synthetase	Smp_104470	3.66	1.00	2.28
ribosomal protein S11	Smp_032760	3.65	1.00	-
alanyl-tRNA synthetase	Smp_038240	3.13	1.00	-
ribosomal protein S2, eukaryotic and archaeal form	Smp_179180	2.80	1.00	2.13
28S ribosomal protein S30	Smp_135810	2.79	1.25	1.00
28S ribosomal protein S5	Smp_145780	2.74	1.00	1.12
ribosomal protein S2	Smp_081960	2.73	1.00	2.14
seryl-tRNA synthetase	Smp_057230	2.67	1.00	1.12
pelota	Smp_002960	2.61	1.00	-
eukaryotic translation initiation factor 3 subunit 5 (M67 family)	Smp_078320	2.61	1.00	2.14
glutamine–tRNA ligase	Smp_148050	2.56	1.00	2.01
zinc finger protein	Smp_095290	2.48	1.05	1.00
eukaryotic translation initiation factor 3	Smp_191990	2.48	1.00	-
methionine-tRNA synthetase	Smp_040770	2.42	1.00	-
elongation factor 1-gamma	Smp_083410	2.40	1.00	1.78
aspartyl-tRNA synthetase	Smp_041450	2.33	1.00	1.96
DNA polymerase theta	Smp_155500	2.31	1.21	1.00
60S acidic ribosomal protein P0	Smp_009690	2.30	1.00	-
60S ribosomal protein L7a	Smp_177210	2.17	1.00	-
60S ribosomal protein L4	Smp_153790	2.17	1.00	1.84
eukaryotic translation initiation factor 3	Smp_000480	2.16	1.00	-
tyrosyl-tRNA synthetase	Smp_129210	2.16	1.00	2.36
seryl-tRNA synthetase	Smp_053610	2.12	1.00	1.95
glutamyl-tRNA synthetase, cytoplasmic	Smp_138930	2.05	1.00	-
60S ribosomal protein L3	Smp_047200	2.04	1.00	1.84
60S ribosomal protein L13	Smp_022640	1.97	1.00	1.79
glutamine synthetase 1, 2	Smp_091460	1.00	5.17	2.00
glycyl-tRNA synthetase	Smp_040800	1.00	2.81	1.40
eukaryotic translation initiation factor 2c	Smp_102690	1.00	2.33	1.23

#### Protein Glycosylation

**Figure 1:**
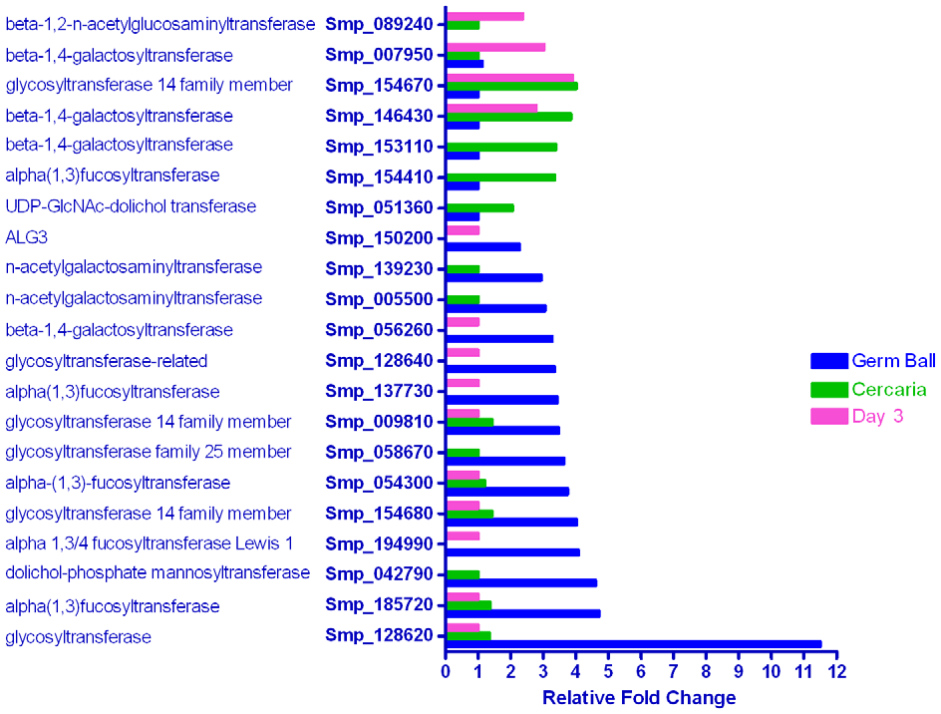
Genes encoding glycosyl transferases. In this and the subsequent figures the relative levels of transcription are shown, with the stage having the lowest expression set to unity. Each locus is labeled with its systematic identity and gene product. Of the 87 glycosyl transferases in the *S. mansoni* genome, 21 were differentially expressed in the three life cycle stages studied. The majority of the up-regulated glycosyl transferase genes were in the germ ball (14/21), with fewer in the cercaria and day 3 schistosomulum (five and four, respectively). This correlates with the known high levels of protein glycosylation of the glycocalyx and gland cell secretions of the cercaria.

Excluding the enzymes of glycogen metabolism, and concatenated gene models, the *S. mansoni* genome encodes at least 87 glycosyl transferases and associated enzymes, according to Pfam and Interpro annotations. Apart from a single xylosyl transferase, the rest are present in multiple copies ranging from two (oligosaccharyl transferase) to 15 (α 1,3 fucosyl transferase). In the glycosyl transferase category, 21 genes were differentially transcribed (Figure 1), primarily enriched in the germ ball (14), and cercaria (5), with two (a galactosyl transferase and a beta-1,2-n-acetylglucosaminyltransferase) up-regulated in the day 3 schistosomulum. The enzymes range across the whole spectrum of reactions involved in glycan synthesis. Thus, for N-linked sugars, a UDP-N Acetyl glucosamine-dolichol phosphate transferase and a dolichol phosphate mannosyl transferase act upstream of ALG3 that transfers mannoses to the nascent branched glycan chain, were up-regulated in the germ ball or cercaria. For O-linked glycans, two N-acetylgalactosamine transferases that initialize formation are germ ball-enriched.

Distal to these initial steps of N and O glycan synthesis, and largely in the Golgi apparatus further transferases extend the sugar chain. We infer that the germ ball-enriched glycosyl-transferases decorate the secreted proteins of the cercarial acetabular glands [35] and are involved in formation of the prominent 1 µm thick glycocalyx [36] by the tegument. The water-proofing provided by the mucin-like glycocalyx is revealed when its shedding, during entry to the skin, renders the parasite vunerable to osmotic stress. The enrichment of five α-1,3-fucosyl transferases (one third of the genome complement) coincides with the presence of oligofucosyl appendages attached to N-acetylated hexose backbones in both N-linked and O-linked glycan structures characterised on acetabular gland proteins [35] and in the O-linked glycans of the structurally complex glycocalyx [36]. Three germ ball-enriched fucosyl transferases (Smp_185720, Smp_054300 and Smp_137730) were also found to be up-regulated in intramolluscan stages by Fitzpatrick *et al.* [13]. Similarly, β-1,4-galactosyl linkages, catalysed by three galactosyl transferases, are found in the same structures, while three closely related glycosyl transferase family 14 members are responsible for β-1,6 linkage of N-acetyl glucosamines at branch points of O-linked glycans. Such structures have been described in the secretions [35] and glycocalyx of the cercaria [36]. The potent immunogenicity of the larval glycans is well documented [37]–[39] but their significance for parasite-host interactions is controversial. Many of the salient features are shared by egg glycans, but use of live eggs as immunogens fails to protect mice against cercarial challenge [37]. Indeed the intensity of the anti-glycan response has led to the suggestion that these structures represent a smokescreen to deflect antibodies from binding key functional epitopes [39], or as a ‘matador's cloak’ [35] whereby the invading larva decoys leukocytes to secretions, whilst it moves stealthily away.

#### Energy metabolism

**Figure 2:**
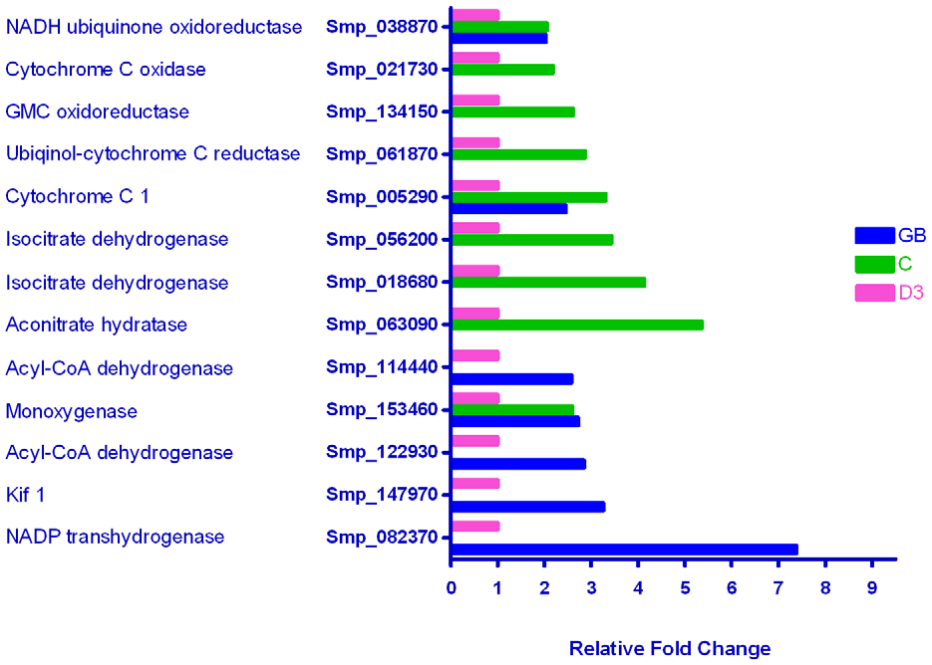
Genes whose products are involved in aerobic metabolism. The relative expression levels of the thirteen differentially expressed genes encoding proteins involved in aerobic metabolism are shown. The majority (9/13) were up-regulated in the cercaria, with 7 in the germ ball, and none in the day 3 schistosomulum. This reflects the intensive energy requirements of the rapidly swimming cercaria.

In spite of the parasite transition from snail hepatopancreas via fresh water to mammalian skin, i.e. low to high and back to low oxygen tension, only a small number of genes classified by GO as associated with aerobic processes were up-regulated in the germ ball or cercaria (Figure 2). These included acyl CoA dehydrogenase required for the cycle of fatty acid oxidation, 13 members of the citric acid cycle e.g. isocitrate dehydrogenase and the electron transport chain e.g. cytochrome c reductase. Of the 30 genes whose products were involved in glycolysis, only lactate dehydrogenase [40] showed a marked (14 fold) increase in transcription in the day 3 schistosomulum (Table S2). Citric acid cycle enzyme transcripts were consistently up-regulated in the cercaria; the highest change was 5 fold (citric acid cycle enzyme aconitase). This corroborates the findings of Skelly *et al.* who showed that cercariae use aerobic metabolism, whereas schistosomula relied on glycolysis to supply energy [6], [41]. Given the extreme rapidity with which the cercarial tail oscillates and its limited glycogen store, the use of aerobic metabolism to yield the maximum possible molecules of ATP is optimal. However, the phosphorylase enzymes involved in glycogen mobilisation were not up-regulated.

#### Lipid metabolism

Lipid metabolism might be a target for interventions, due to the dependence of schistosomes on the host for supply of basic sterols and fatty acids [16]. The *S. mansoni* genome paper [16] listed 102 schistosome genes as playing a role in lipid synthesis, transport or degradation, 23 (22.5%) of which were differentially transcribed (excluding the saposins; Table 3). The five genes enriched in the germ ball (3 to 6 fold) were all involved in synthesis of complex lipids, those in the cercaria and day 3 schistosomulum in acquisition, synthesis, and degradation. The enrichment of two phospholipases in the day 3 schistosomulum may be evidence for autophagy coincident with body remodeling. More than 50% of the differentially transcribed genes involved in lipid metabolism were up-regulated in the cercaria. They represent a heterogeneous assemblage, some potentially associated with the development of the gut (NPC1 and Saposins; see below) and others with signalling pathways. Sphingosine kinase is required for the formation of sphingosine 1 phosphate, which can act in both intra- and extra-cellular signalling. Conversely, inositol monophosphatase reduces the signalling activity of phosphoinositides controlling calcium release from the sarcoplasmic reticulum, and hence muscle contraction. Finally, by analogy with the stratum corneum of the skin [42], the up-regulation of ceramide synthase could serve an osmoprotective function if its product were localised in the tegument surface.

**Table 3:** Lipid metabolism.

Annotation	Gene ID	GB	C	D3
elongation of fatty acids protein 1	Smp_051810	5.82	1.00	-
mevalonate kinase	Smp_042980	4.05	1.00	-
elongation of fatty acids protein 1	Smp_130370	3.12	1.00	1.07
serine palmitoyltransferase 2	Smp_079380	2.89	-	1.00
Lamin B receptor (ERG24)	Smp_124300	2.70	1.11	1.00
inositol monophosphatase	Smp_095000	-	2.20	1.00
acetyl-CoA carboxylase	Smp_123710	-	2.23	1.00
niemann-pick C1 (NPC1)	Smp_039130	1.00	2.66	-
hormone-sensitive lipase (S09 family)	Smp_000130	1.00	2.90	-
sphingoid long chain base kinase	Smp_157100	1.00	3.48	1.72
lipase 1	Smp_146180	1.69	3.60	1.00
sphingosine kinase	Smp_117370	1.00	3.94	-
choline kinase	Smp_015050	1.09	5.86	1.00
FFA transport protein, putative	Smp_143830	1.00	6.29	-
(dihydro)ceramide Synthase (LAG1)	Smp_042440	-	7.94	1.00
diacylglycerol O-acyltransferase 1	Smp_127280	1.86	14.33	1.00
3-keto-dihydrosphingosinereductase	Smp_141720	-	1.00	2.14
hydroxymethylglutaryl-CoA synthase	Smp_155270	1.85	1.00	2.38
inositol transporter	Smp_134080	1.00	-	3.38
fatty acid acyl transferase-related	Smp_010770	-	1.00	5.50
phospholipase D	Smp_151420	1.00	2.09	6.20
sterol O-acyltransferase 1	Smp_134390	1.32	1.00	6.30
phosphatidylcholine-sterol acyltransferase	Smp_031180	1.00	1.23	13.93

#### Defence against stress

On the assumption that larval schistosomes experience stress during the mammalian infection process, the expression patterns of stress-related genes were investigated. Of the 29 genes in this category, six were differentially transcribed (21%; Table S3). None were germ ball-enriched, while those encoding universal stress protein, glutathione peroxidase and a multidrug resistance pump (MDR; Smp_151290) were up-regulated in the cercaria. Also notably up-regulated at this stage was a small heat shock protein, Sm-p40 (18 fold), which comprises 15% of the soluble proteome in the other fresh water stage, the miracidium [43]. This chaperone may protect proteins from irreversible aggregation upon stress-induced denaturation, in an energy independent manner [43]. Another MDR (Smp_135490) was up-regulated in the day 3 schistosomulum, along with a superoxide dismutase and a thioredoxin peroxidase (37 fold higher than in the germ ball). This protein, first cloned from adult worms [44], has been reported in acetabular gland secretions [45] and its up-regulation in the schistosomulum suggests an enhanced role in the migratory parasite possibly in defence against oxidative attack from phagocytes. The transcript of the proposed anti-inflammatory protein Sm16 [46] was germ ball-enriched. The protein was abundant in cercarial secretions [2] and has been variously reported as an immunomodulator [47], “stathmin-like” [48], and more recently as an inhibitor of cytokine production in whole human blood cultures, via its ability to block TLR signalling [49]. Its role remains enigmatic.

### Parasite tissues

This category encompasses the mechanisms involved in the development and differentiation of tissues and the transcription of genes linked to three specific tissues, namely muscle, gut and tegument.

#### Development of tissues

**Figure 3:**
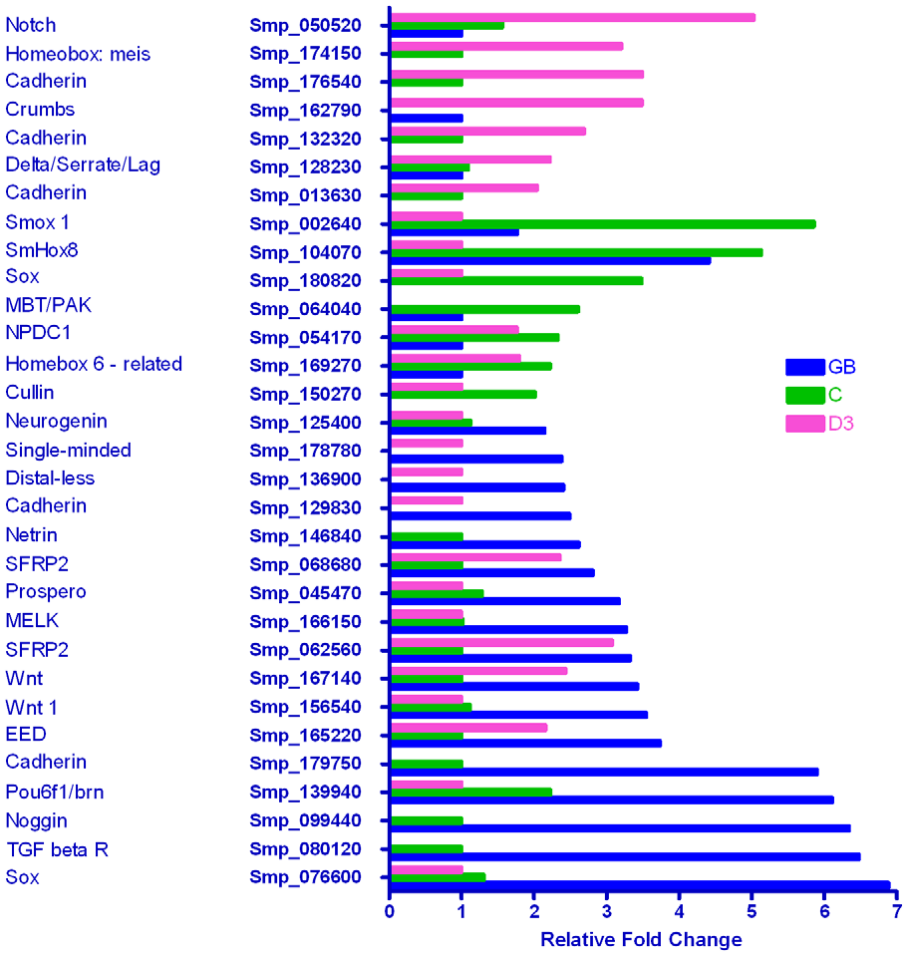
Genes whose products are involved in development. More than half of the differentially expressed developmental genes were enriched in the germ ball (18/31). In the cercaria, 8/31 were up-regulated, and in the day 3 schistosomulum 11/31. Four genes were up-regulated in both the germ ball and schistosomulum compared to the cercaria, while two were enriched in the germ ball and cercaria compared to the schistosomulum. These data highlight the differences in developmental processes occurring in each of the larval stages.

A total of 31 genes out of 158 in the custom development category were differentially transcribed in one or more life cycle stage (Figure 3). More than half of these (17/31) were up-regulated in the germ ball, fewer in the cercaria (7/31) or day 3 schistosomulum (6/31). Of the nine genes involved in neural development, four were transcribed most highly in the germ ball, two in the cercaria and three in the day 3 schistosomulum. The early neural patterning genes, ‘sox-like’ transcription factor, maternal embryonic leucine zipper kinase (MELK), ‘single-minded’ and ‘neurogenin’ were notable in the germ ball. The basic layout of the central ganglia and nervous system is developed by the time the germ ball reaches the stubby tailed stage [50]. Although the extent of these ganglia increases during intra-mammalian life, their position remains unchanged.

The polarity complex component ‘Mbt/PAK’ was up-regulated in the cercaria while ‘stardust’ and two ‘notch’ genes were enriched in the day 3 schistosomulum. Four genes, embryonic ectoderm development protein, Wnt 1 (Smp_167140) and two secreted frizzled-related protein 2 (SFRP2) Wnt inhibitors were highly transcribed in the germ ball and day 3 schistosomulum, compared to the cercaria. A second Wnt 1 gene (Smp_156540) was up-regulated solely in the germ ball, and a Hox 8 in both the germ ball and cercaria compared to the day 3 schistosomulum. These genes control dorso-ventral and anterio-posterior patterning [51]–[53], essential processes during embryogenesis. The schistosome orthologue of the transforming growth factor beta receptor (TGFβR), but not its potential endogenous ligands Smp_063190 and Smp_146790, was up-regulated six fold in the developing germ balls compared to the cercaria, as might be expected for a protein that controls proliferation and cellular differentiation.

A total of 26 cadherins, proteins involved in cell-cell adhesion, are present in the *S. mansoni* genome. Two transcripts were up-regulated in the germ ball, while three different ones were enriched in the day 3 schistosomulum, suggesting specific requirements for the association of different cell types. There was a similar variability in the homeobox genes transcribed in each stage: germ ball ‘prospero’ and ‘distal-less’; cercaria ‘homeobox six-related’, ‘Hox8’, and ‘Smox1’; schistosomulum, ‘meis’. Among the seven cercaria-enriched genes, were Neural proliferation differentiation control 1 (NPDC1), and cell polarity protein, which could play a role in photoreceptor cell morphogenesis. Although the cercaria is the least translationally active stage, it is transcribing a battery of genes involved in developmental processes as a precursor to post-infection tissue changes. A number of genes involved in developmental processes are also enriched in the day 3 schistosomulum, distinct from the germ ball and cercaria; these include tolloid, delta/serrate/lag, and notch. The absence of cell division at this stage suggests such genes are active pending arrival of the schistosomulum in the portal tract.

#### Muscle and Actin binding proteins

The muscles of the cercarial tail, responsible for the extremely rapid (22/sec) propulsive oscillations [54] have a structure akin to that of the sarcomeres in the striated muscle of higher animals [55], with a pseudostriated appearance. There are regular arrays of transverse sarcoplasmic reticulum interdigitating with dense bodies, equivalent to the Z line of striated muscle, to which the actin filaments are attached [55]. We anticipated that the unique organization of cercarial tail muscle would be reflected in the expression patterns of genes encoding muscle proteins. A search using the GO term ‘Actin binding’, which comprises 330 *S. mansoni* genes, revealed a small differentially transcribed subset, 20 of which were up-regulated in the cercaria (2 to 30 fold) (Table 4). A PDZ/LIM domain-containing orthologue of mammalian cypher was the most highly transcribed. PDZ domain proteins play an important role in organising protein networks on membranes, while the zinc binding LIM domain is also involved in cytoskeletal adhesion. Other prominent genes encoded: an alpha actinin, which binds actin to z-lines; proteins containing IQ domains, extremely basic motifs which are binding sites for EF hand proteins, including myosins and calmodulins, suggesting a role in muscle contraction; FH2/FH3 domains (a formin); three 4.1 G proteins. Formins play a pivotal role in the organisation of the actin cytoskeleton. Their FH2 domain is required for self-association and the FH3 domain for intracellular localisation; it contains a GTPase binding site indicative of signalling processes. The 4.1 G proteins contain FERM domains, which localize proteins to the plasma membrane. Other cercarial-enriched transcripts included one of the nine titins in the genome, one of the 14 dynein light chains and the single copy genes for myosin light chain and coronin.

**Table 4:** Actin binding and muscle proteins.

Annotation	Gene ID	GB	C	D3
cypher orthologue	Smp_157310	1.94	30.25	1.00
dynein light chain	Smp_174510	1.00	20.99	-
alpha-actinin	Smp_034550	1.00	9.53	1.00
4.1 G protein	Smp_095620	1.00	7.34	-
fh1/fh2 domains-containing protein	Smp_161110	1.00	4.41	-
4.1 G protein	Smp_125440	-	3.93	1.00
4.1 G protein	Smp_018670	1.00	3.77	-
calponin/transgelin	Smp_126480	1.20	3.68	1.00
calponin homolog	Smp_078690	1.00	3.20	2.37
alpha-actinin	Smp_014780	1.00	3.09	-
dynein heavy chain	Smp_160640	1.26	2.88	1.00
calponin-related	Smp_086330	1.00	2.79	-
fimbrin/plastin	Smp_149130	1.00	2.70	1.00
myosin light chain 1	Smp_045220	1.00	2.67	1.00
titin	Smp_105020	1.00	2.63	-
myosin heavy chain	Smp_127460	-	2.58	1.00
coronin	Smp_012720	1.00	2.52	1.08
troponin t, invertebrate	Smp_179810	1.00	2.41	2.02
ferm domains	Smp_149640	-	2.19	1.00
tropomodulin	Smp_003710	1.00	2.00	-

#### Alimentary tract

The transcription patterns of gut-associated genes were scrutinised to discover whether the organ is active in the migrating schistosomulum. Of the 32 genes in this custom category, 20 (60%) were differentially transcribed (Table 5), with 10 of the 12 saposins present in the genome strikingly up-regulated in the day 3 schistosomulum (4 to 36-fold) compared to the germ ball. Five cathepsins and an asparaginyl endopeptidase were similarly up-regulated with up to 50-fold increase compared to the germ ball. Most of these genes were also enriched in the cercaria, the exceptions being a glycosyl hydrolase, cathepsin S, and four of the saposins. Two protease inhibitors, a serpin and alpha 2 macroglobulin were also enriched in the day 3 schistosomulum. The sustained transcription of saposins, first seen in the cercaria, suggest the larval gut is active, potentially acquiring nutrients from ingested plasma before feeding on erythrocytes begins when the portal vein is reached. Indeed, up-regulation of saposins at day 3 is indicative of a much enhanced capacity to acquire lipids, most probably via the gut. The up-regulation of gut proteases is consistent with the immunolocalisation of cathepsin B and asparaginyl endopeptidase to the cercarial gut [56], and the localisation of cathepsin L mRNA to the gut of lung stage schistosomula by WISH [57], indicating that gut-specific proteases are translated and secreted early in infection. The recent oblique demonstration of Lucifer Yellow ingestion by newly transformed schistosomula [58] further reinforces the idea that the early larval gut is active.

**Table 5:** Alimentary tract.

Annotation	Gene ID	GB	C	D3
Cathepsin L	Smp_157090	1.00	2.25	2.39
glycosyl hydrolase	Smp_043390	1.00	-	2.46
serpin	Smp_090080	1.00	3.14	3.12
cathepsin S (C01 family)	Smp_139240	1.00	-	3.81
Saposin containing protein	Smp_028840	1.00	-	4.16
α 2 macroglobulin	Smp_089670	1.00	-	4.20
Saposin containing protein	Smp_137700	1.00	-	5.34
calpain (C02 family)	Smp_157500	1.00	5.32	6.70
Saposin containing protein	Smp_014570	1.00	7.48	6.74
Saposin containing protein	Smp_028870	1.00	-	10.39
Saposin containing protein	Smp_016490	1.00	5.50	10.61
cathepsin B-like peptidase (C01 family)	Smp_103610	1.00	3.04	13.62
saposin containing protein	Smp_105450	1.00	13.22	14.34
Saposin containing protein	Smp_137720	1.00	-	14.55
cathepsin B-like peptidase (C01 family)	Smp_067060	1.00	5.43	15.27
Saposin containing protein	Smp_105420	1.00	5.39	23.45
Asparaginyl endopeptidase (C13 family)	Smp_075800	1.00	13.54	24.62
saposin containing protein	Smp_130100	1.00	-	24.67
Saposin containing protein	Smp_194910	1.00	4.94	36.10
SmCL2-like peptidase (C01 family)	Smp_193000	1.00	8.11	49.87

#### Tegument

Changes in the transcription of genes associated with the tegument were among the most dramatic in this study. This tissue undergoes substantial remodeling on entry to the mammalian host, and is a large component of the host-parasite interface with roles in nutrient uptake, ionic/osmotic balance and defence. The up-regulation of genes encoding tegument proteins may be integral to the large increase in surface area (73% between days 0 and 4) associated with intravascular migration of the schistosomulum to the portal system [59] during which it elongates threefold without an increase in mass [60].

**Figure 4:**
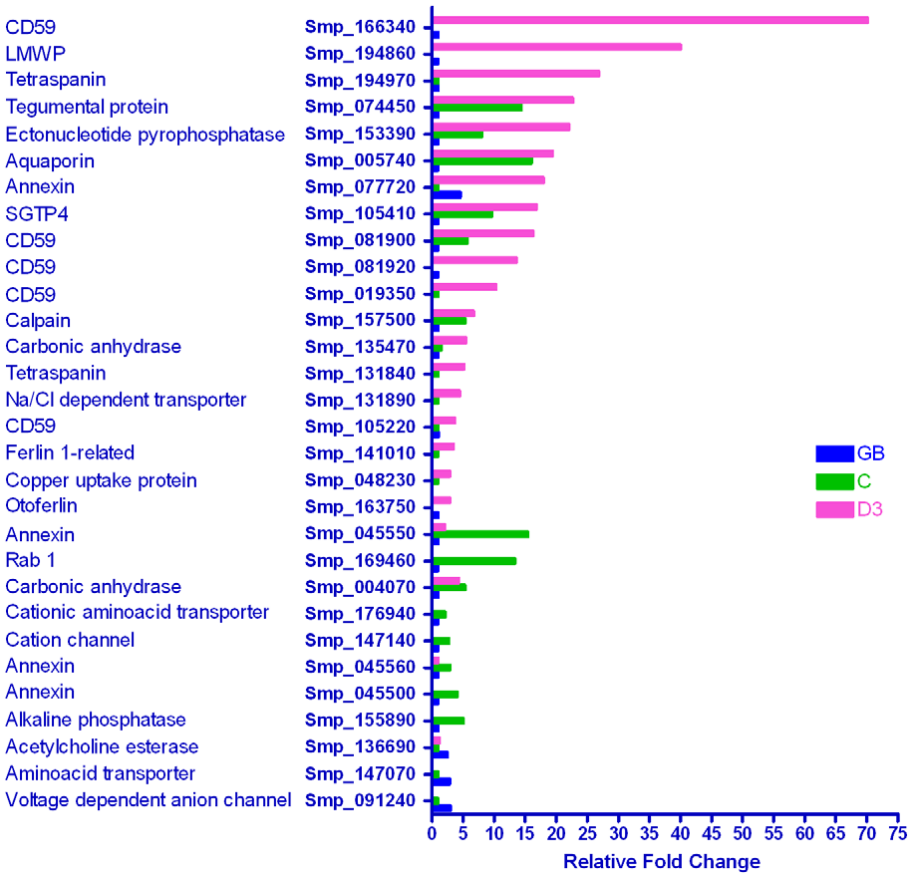
Genes encoding tegument proteins. The genes in this custom category encode proteins that have been identified at the adult worm tegument surface by proteomics or localization studies. Of the 30 differentially expressed genes, 20 were up-regulated in the schistosomulum, 13 in the cercaria and three in the germ ball, with seven up-regulated in both the cercaria and schistosomulum. These data reveal the early transcription of genes involved in remodeling the tegument on parasite entry into the mammalian host, a process that continues thereafter with an orthologue of human CD59 enriched 75 times compared to the germ ball.

Of the 65 genes in the tegument category, only three were most highly expressed in the germ ball (Figure 4). They were a voltage-dependent anion-selective channel, an amino acid transporter, and acetylcholinesterase. In the cercaria, three closely related annexins (e.g. Smp_045550, 15 fold), alkaline phosphatase, a cation channel and a cationic amino acid transporter were most highly transcribed. A single Rab1 membrane GTPase, potentially involved in vesicle trafficking to the cell surface, was also up-regulated in the cercaria compared to the germ ball. As with the alimentary tract, the majority of genes encoding tegument proteins were up-regulated in the day 3 schistosomulum. Five of these encoded copies of a putative complement inhibitor CD59, one of which (Smp_166340) was ∼70 fold higher than in the germ ball. It has not been identified in proteomic analyses of adult worm tegument [27] so its expression may be specific to the schistosomulum. Another CD59 orthologue (Smp_081900) was up-regulated in both the cercaria and the day 3 schistosomulum. In the host, CD59 proteins are self-protective, inhibiting formation of the membrane attack complex C5–C9 of the complement system, and hence cell lysis. The enrichment the *S. mansoni* orthologues at day 3 may represent preparation for intravascular life. Other cercaria and schistosomulum-enriched genes were a calpain (Smp_157500, previously Sm02451 [12]), the glucose transporter SGTP4 (16 fold), an aquaporin (19 fold), an ectonucleotide-pyrophosphatase and a protein of unknown function (Smp_074450), which was discovered on the schistosome surface by Braschi *et al.* [24]. The enzyme phosphodiesterase 5 demonstrated on the surface of adult worms also shares this expression pattern [61], [62]. The aquaporin is of particular note in view of the recent demonstration that it is vital for controlling water and drug flux [63], and lactate excretion [64], across the tegument surface membranes of the adult worm. Transcripts which were up-regulated solely in the day 3 schistosomulum included otoferlin, ferlin, a copper transporter, a sodium/chloride dependent transporter and two tetraspanins. The tetraspanin Smp_194970 has been found previously (was Sm04463 [13]). Sm8.7 (low molecular weight secreted protein, LMWP) was strikingly up-regulated (40 fold compared to the germ ball) and merits a deeper investigation. It lacks a membrane anchor and has no putative domains or homology to proteins outside the Class Trematoda. It was first described from *Clonorchis sinensis*, is present in *S. japonicum* [65] and in *Fasciola hepatica* (unpublished data); its ubiquity suggests a trematode-wide commonality of function.

Transcription of the known tegumental carbonic anhydrase (Smp_168730), hypothesised to facilitate CO_2_ export from the worm [61], did not vary, but two other carbonic anhydrase enzymes were differentially regulated, one enriched in the cercaria (Smp_004070; 5.3 fold), and the other in the day 3 schistosomulum (Smp_135470; 5.5 fold). It is important to determine whether either or both of these enzymes are tegument-associated as they may act in concert with two anion exchange channels (see membrane: channels category) to deal with the high CO_2_ concentration in the dermal tissues.

### Molecular function

This category encompasses groups of genes either with a similar organization (the MEGs) or those that encode proteins with a related molecular structure or function.

#### Proteases: General

A total of 335 proteases were annotated in the *S. mansoni* genome. Appreciable numbers belonging to four groups, based on their MEROPS database assignments, were found to be differentially regulated. No proteases belonging to the G (glutamic acid) or U (Unknown catalytic site) classes, and only two T (threonine) proteases were detected. One of these, a proteasome subunit (Smp_070930), was up-regulated in the germ ball, while the other, involved in degradation of glycoproteins (Smp_173480), was up-regulated in both the germ ball and day 3 schistosomulum.

#### Proteases: Serine

**Figure 5:**
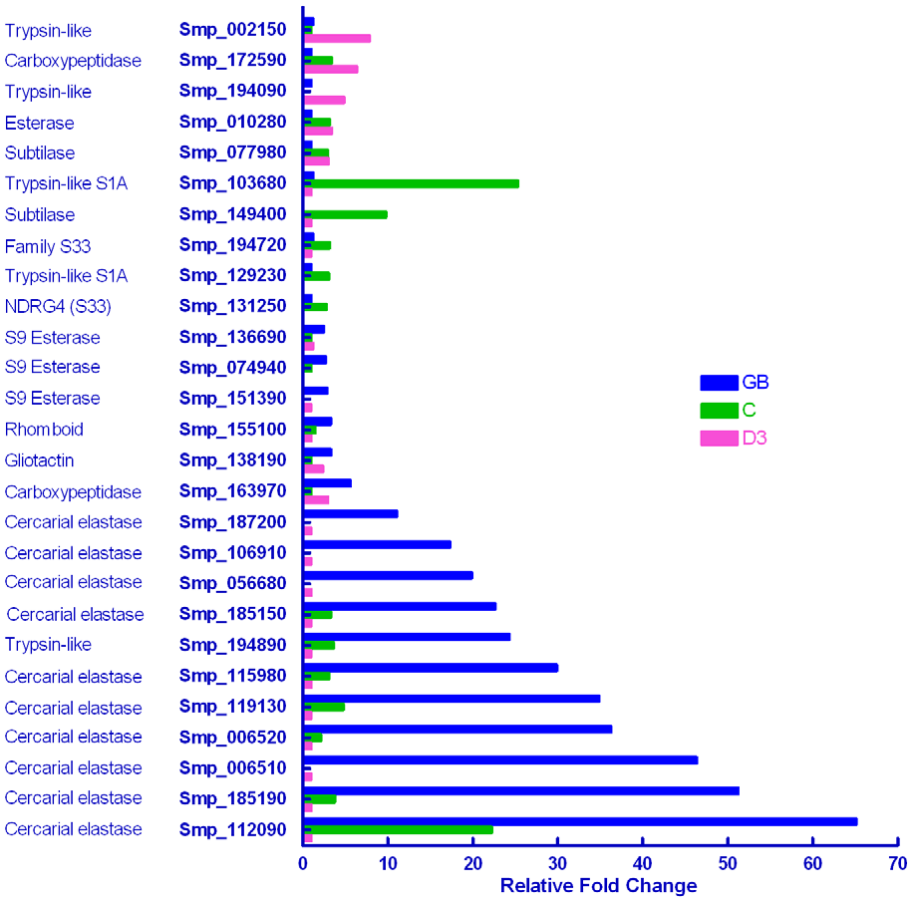
Genes encoding serine proteases. The differential expression patterns of 27 of the 78 serine proteases encoded in the *S. mansoni* genome. Seventeen were up-regulated in the germ ball, of which 10 encoded cercarial elastases, expressed 10–65-fold higher than in the day 3 schistosomulum. Eleven serine proteases were enriched in the cercaria, and five in the schistosomulum. The study reveals a wider range of cercarial elastases potentially involved in skin invasion than hitherto reported.

Of the 78 serine proteases annotated in the genome, 27 (35%) were differentially transcribed (Figure 5). They included 11 genes encoding cercarial elastase (a constituent of acetabular gland secretions) that were 10–65 fold more highly expressed in the germ ball. Previously, Salter *et al.* described five cercarial elastase genes, stating that two of them accounted for 90% of the protein detected [66]. Also up-regulated in the germ ball are three family S09 esterases (non-peptidase homologues), one family S10 carboxypeptidase, gliotactin, and a transmembrane rhomboid protease. Rhomboid proteases have been implicated in cell invasion by *Toxoplasma* [67] and *Plasmodium* [68], immune evasion by *Entamoeba histolytica* [69], and in cell signalling in *Drosophila* [70]. It is unclear whether inhibition of rhomboid proteases can prevent invasion by parasites, and furthermore whether they are suitable drug targets [70]. Five serine proteases were up-regulated in the cercaria, with 2 to 5 fold changes. Two may have a role in cell differentiation (S33 family), one is a subtilase, and the other two belong to the S01 family (trypsin/chymotrypsin). The remaining five differentially transcribed serine proteases, up-regulated in the cercaria and schistosomulum, included a different S08 subtilase and a S09 esterase. Two of the genes up-regulated in the day 3 schistosomulum alone were a carboxypeptidase (family S10) and, in keeping with previous work, the trypsin-like Smp_002150 which has homology to Antigen 5 from *Echinococcus granulosus* (was Sm12764; [12]).

#### Proteases: Metallo

**Figure 6:**
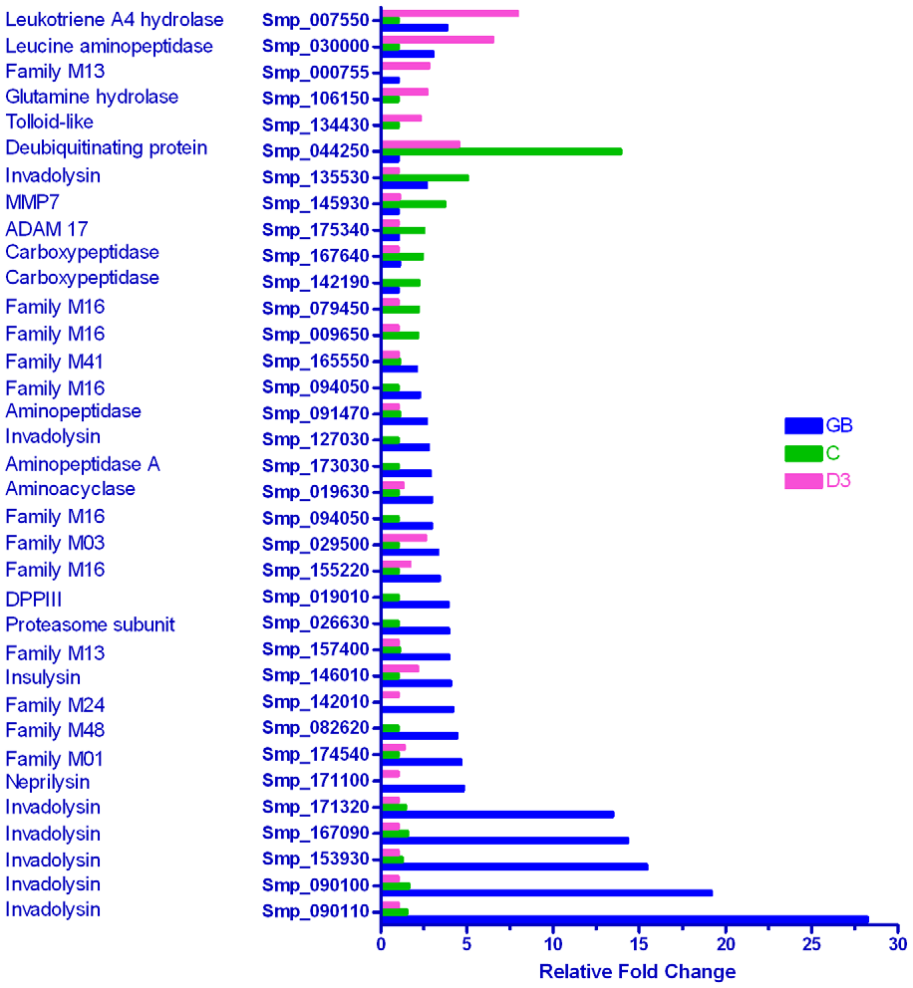
Genes encoding metalloproteases. The majority (22/35) of differentially expressed metalloprotease genes were up-regulated in the germ ball. This includes six invadolysins with expression levels ranging from 2 to 27-fold higher than either of the other life cycle stages. By contrast, only seven genes were up-regulated in the schistosomulum, the highest fold change being ×10. These observations strongly implicate a range of metalloproteases in the skin invasion process.

Of the 114 authentic metalloproteases described in the genome paper, 35 (31%) were differentially expressed, the majority (22/35) again being enriched in the germ ball (Figure 6). The five most highly up-regulated (13–28 fold) were all invadolysins; a sixth (Smp_127030) was transcribed 2.2 fold higher and a seventh (Smp_135530) was five-fold higher in the cercaria compared to the other two stages. The leishmanolysin described by Curwen *et al.* (Smp_090100) [2] was the first metalloprotease identified in a proteomic analysis of cercarial secretions and is now annotated in the genome as an invadolysin. The remaining germ ball-enriched metalloproteases are involved in translation and cell proliferation. Two mitochondrial processing peptidases, two carboxypeptidases, an ADAM protease (a disintegrin and metalloprotease), a matrix metalloprotease (MMP7) and a proteasome regulatory subunit (14 fold higher than the germ ball) were up-regulated in the cercaria. Five metalloproteases were up-regulated in the day 3 schistosomulum, including tolloid-like protease, leucine aminopeptidase, possibly associated with enhanced gut function, and glutamine hydrolase. Our observations suggest a greatly expanded repertoire of proteases potentially involved in infection by larval schistosomes. Indeed, we might ask why the cercaria needs so many closely related proteases. Do they have subtly different substrate targets, or do they represent immune evasion by diversification? It is plausible that antibodies capable of neutralising the activity of one or more elastases or invadolysins may not be effective against all of them.

#### Proteases: Cysteine

**Figure 7:**
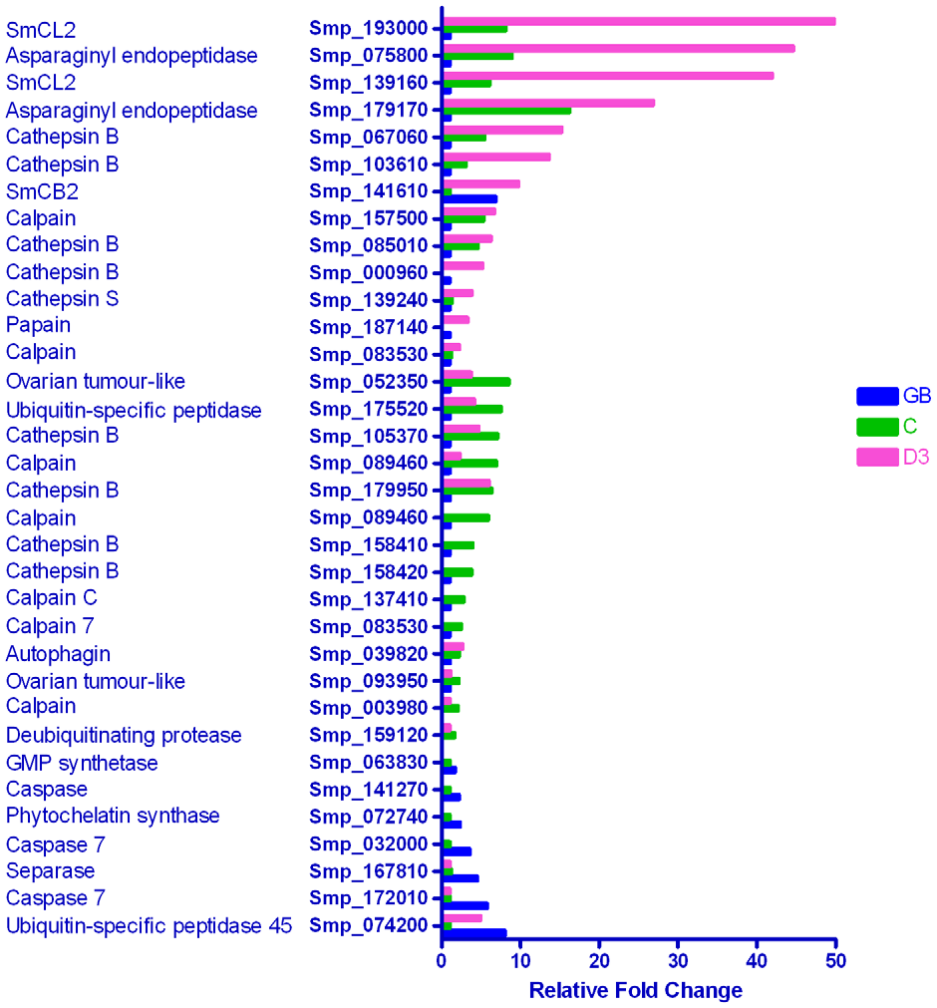
Genes encoding cysteine proteases. The majority of cysteine protease genes were up-regulated in the schistosomulum, the fold changes for three being >40×, with fewer in the cercaria and germ ball. We infer that the up-regulation seen in the schistosomulum is related to the early differentiation of the parasite gut.

Of the 97 cysteine proteases annotated in the *S. mansoni* genome, 34 (35%) were detected as differentially expressed in this study (Figure 7). In contrast to the metallo- and serine- proteases, they were largely up-regulated in the cercaria and/or day 3 schistosomulum, the highest fold changes being in the latter. The seven germ ball-enriched cysteine proteases, three of which are caspases, displayed 2 to 7.5 fold changes compared to the cercaria, whilst a ubiquitin-specific protease was up-regulated in both the germ ball and the day 3 schistosomulum. The remaining germ ball-enriched transcripts encoded a separase (caspase-like protein involved in cell division), a GMP synthase, and a phytochelatin synthase, involved in the synthesis of a heavy metal binding protein, and hailed as a potential drug target [71].

Five calpains, four cathepsin B-like proteases, two deubiquitinating enzymes, two genes with homology to the *Drosophila* ovarian tumour gene and an autophagin were most highly transcribed in the cercaria. In the first 3 days after cercarial transformation, remodelling of the body occurs in the absence of cell division. This involves complete disappearance of the acetabular glands (∼25% percent of the body volume, [19]), and repositioning of the schistosomulum musculature, with loss of the subtegumentary fibrous interstitial layer to accommodate the enormous capacity for body extension [72]. Given the extent of this remodelling, we might anticipate the up-regulation of genes encoding proteins involved in apoptosis, autophagy, or intracellular protein degradation by the proteasome pathway. Surprisingly, these were not evident, perhaps due to the earlier timing of these events. Indeed, the cercarial enrichment of many of the genes listed above, plus ADAM and MMP7 in the metalloprotease category, suggests that genes encoding proteins involved in remodelling are transcribed early in preparation for host invasion.

Two calpains, eight cathepsins, and two asparaginyl endopeptidases were most highly transcribed in the schistosomulum. They show striking increases, up to 50 fold, with seven being up at least 10 fold relative to the germ ball, thus sharing the expression pattern of known gut-associated genes. Only one cathepsin B-like gene (Smp_141610) was up-regulated in both the germ ball and day 3 schistosomulum compared to the cercaria.

#### Proteases: Aspartic

This fourth group of proteases has homology to the acidic digestive enzymes Cathepsin D and pepsin. All were dominant in the day 3 schistosomulum with levels ranging from 2 to 15 fold higher than in the germ ball, sharing the expression pattern of known gut-associated genes. Three genes, were also 2 to 4 fold higher in the cercaria than the germ ball (Table S4). One such aspartic protease activity (Smp_013040) has been reported from schistosomula [73]. The identification of the eight aspartic protease transcripts up-regulated in the day 3 schistosomulum (not including Smp_013040) greatly expands the repertoire of enzymes potentially involved in digestion.

#### Membranes: Overview

Membrane proteins which cannot be assigned to a specific tissue, are described in detail below, grouped by putative function. The GOstats analysis highlighted membrane proteins up-regulated in the cercaria and day 3 schistosomulum, but not in the germ ball.

#### Membranes: Transporters

In the cercaria, three sodium-neurotransmitter symporters were up-regulated along with another sodium-dependent transporter and a monocarboxylate transporter (Smp_151010; 4.5 fold, Table S5). The three genes encoding sodium neurotransmitter symporters may be involved in neural activity, potentially related to cercarial swimming and host location behaviour. The day 3 schistosomulum transcribed a different suite of transporters. Of these, two amino acid transporters (Smp_176930, and Smp_123010, the latter cationic), distinct from that already mentioned in the tegument category, showed the greatest change (19 and 6 fold) compared to the germ ball. Their enrichment may also be indicative of tegument surface reorganisation to facilitate nutrient uptake. In particular, Smp_176930 has 45% identity, 65% conserved amino acids with the known amino acid transporter of the adult tegument (Smp_176940), the two genes being adjacent on a chromosome. This may indicate a degree of stage-specificity in amino acid uptake. Also enriched in the day 3 schistosomulum were a second monocarboxylate transporter (Smp_146830; 6 fold), three zinc transporters, two catecholamine transporters, a mitochondrial glutamate carrier and glycerol-3-phosphate transporter. If the zinc transporters are located at the tegument or gut surfaces, their expression presumably secures a supply of this important cofactor required for enzyme activity (e.g. of carbonic anhydrase or carboxypeptidase). A Na-dependant bile transporter was up-regulated in the cercaria and day 3 schistosomulum. The majority of bile salts in the intestine are actively reabsorbed into the hepatic portal circulation [74] where they can be ingested by schistosomes. Hence this membrane transporter could acquire the essential dietary sterols. A stomatin (Smp_003440, one of nine in the genome), was up-regulated in the germ ball and schistosomulum. The human orthologue of this protein is highly expressed in the surface membrane of erythrocytes (band 7 protein), where it is associated with lipid rafts and microdomains.

#### Membranes: Channels

Channels are important for allowing substrates to pass into and out of cells and organelles; as such they may be expressed at the host-parasite interface. An amiloride-sensitive sodium channel was transcribed 8 fold higher in the cercaria, compared to the day 3 schistosomulum (Table S6). This channel has two potential functions, depending on its site of expression. Its nearest homologues outside the schistosomes are the FMRFamide-gated channels of molluscan neurones [75] where it serves an excitatory role. It is noteworthy that FMRFamide-related peptides have been shown to contract schistosome muscle [76] implying the existence of a receptor. Alternatively, amiloride-sensitive sodium channels in vertebrates are located at the luminal surface of transporting epithelia, and responsible for maintaining salt and water balance. The up-regulation of a homologue (Sm13225, not in genome assembly) in the lung schistosomulum was noted in an earlier microarray experiment [12]. Which of these roles is played by the cercarial channel remains to be investigated. Similarly, polycystin, an unusual cation channel associated with monogenic polycystic kidney disease in humans, was up-regulated four fold in the cercaria relative to the schistosomulum. Polycystin (PKD2) may play a role in the context of chemoreception. In *C. elegans*, PKD2 protein localises to the cilia of sensory neurons; mutations in the *pkd2* gene are linked to defects in chemo- and mechanoreception by the worm [77]. This is highly relevant to the *S. mansoni* cercaria in view of the ubiquity of cilia on its numerous sensory endings [78].

Potassium channels were up-regulated in both the cercaria and the day 3 schistosomulum. The cercarial enrichment of weakly inward-rectifying potassium channels (TWIK family), which maintain membrane potentials, may reflect the intense muscular activity of this stage. The up-regulation of four voltage-gated potassium channels in the day 3 schistosomulum could be linked to the marked changes in rhythmic muscular activity associated with intravascular migration [79]. Two anion exchange channels were up-regulated in the cercaria (Smp_180950) and schistosomulum (Smp_136030), respectively. They are noteworthy because they appear to exchange chloride for carbonate ions. The analogous protein in humans is located at the erythrocyte surface and is responsible for the chloride shift during CO_2_ transport. A single mitochondrial import receptor which forms channels in the mitochondrial outer membrane, was up-regulated in the day 3 schistosomulum.

#### Membranes: Receptors

At least 92 G-Protein coupled-receptors (GPCRs) are encoded in the *S. mansoni* genome but relatively few were differentially transcribed across the three lifecycle stages. These genes were almost exclusively up-regulated in the day 3 schistosomulum (Table S7). However, the 9 fold enrichment of an opsin family GPCR in the germ ball, is of particular note because of the well-documented phototaxis of the schistosome cercaria. To our knowledge no convincing structure with the morphology of an eyespot has ever been described in the cercaria; the localisation of this GPCR by whole-mount *in situ* hybridisation (WISH) could provide an appropriate pointer. A muscarinic acetyl choline receptor was enriched in the cercaria. A conserved domain search of this GPCR (Smp_145540) identifies it as a potential serpentine type chemoreceptor (srsx; cf. *Caenorhabditis elegans*). As such it is a prime candidate for transduction of the chemical cues used by the cercaria to locate its mammalian host.

In the day 3 schistosomulum five GPCR genes were up-regulated; these encoded single members of the rhodopsin-like orphan, muscarinic acetylcholine (Smp_152540, also found by Fizpatrick *et al.* [13]), beta peptide, alpha biogenic amine, and ionotropic glutamate classes. Such GPCRs mediate diverse aspects of neurotransmission and their expression may be related to the reorganisation of the nervous system after host entry, e.g. loss of the sensory papillae and pits present on the apical area of the cercaria. The up-regulation of two catecholamine transporters may also be relevant to these changes. A further four non G-protein coupled receptors are also up-regulated in the day 3 schistosomulum. Two of these, annotated as adiponectin receptor and progestin/adipoq receptor, share 26% identity and 42% positive amino acids. Their up-regulation in the larval stage about to enter the blood stream is intriguing in view of the presence of host adiponectin, a hormone that modulates processes such as glucose regulation and fatty acid catabolism, at high concentration in the circulation. Whether these schistosome receptors can respond to host adiponectin or progesterone remains to be investigated. However, BLAST searching of the NCBInr database with vertebrate adiponectin protein sequences, trimmed of their collagen domains, reveals two potential endogenous ligands, Smp_105050 and Smp_023240, that share 28% identity and 46% conserved amino acids with vertebrates over the key regions of sequence and 47% identity and 67% conserved amino acids with each other. The transcription levels of the potential endogenous ligands were low and neither changed across the three lifecycle stages. A CD36 orthologue and a P2X receptor were also up-regulated in the day 3 schistosomulum. The former is characterised in the mammalian host as a scavenger receptor interacting with numerous ligands. The latter has been cloned and characterised [80], [81]. P2X receptors are cation-permeable channels that open in response to extracellular ATP. It remains to be clarified whether the schistosomulum P2X receptor utilises an endogenous purinergic signalling system or responds to host-derived ATP. Localisation of this receptor within the parasite is thus of paramount importance.

#### Membranes: Structural proteins and enzymes

Two ATPases, (one a calcium-transporting sarcoplasmic reticulum type and the other a flippase) were up-regulated in the cercaria. The remaining differentially transcribed genes encoding membrane proteins were up-regulated in the day 3 schistosomulum (Table S8). The up-regulation of membrane structural ferlins and six tetraspanins, although not definitively tegument-associated, may be related to the increased tegument surface area and to changes in membrane structure; in particular tetraspanin Smp_059530 (67-fold), would repay further investigation as it could be unique to larval membranes. The up-regulation of Smp_194980 and Smp_041460 (formerly Sm12883), is in keeping with previous work [12], [13]. The presence of three innexins and an integrin, further emphasises the importance of cell-cell interactions in the day 3 schistosomulum. A transcript encoding peptidyl-glycine alpha-amidating monooxygenase was enriched in the day 3 schistosomulum.

#### Venom allergen-like proteins (VALs)

**Figure 8:**
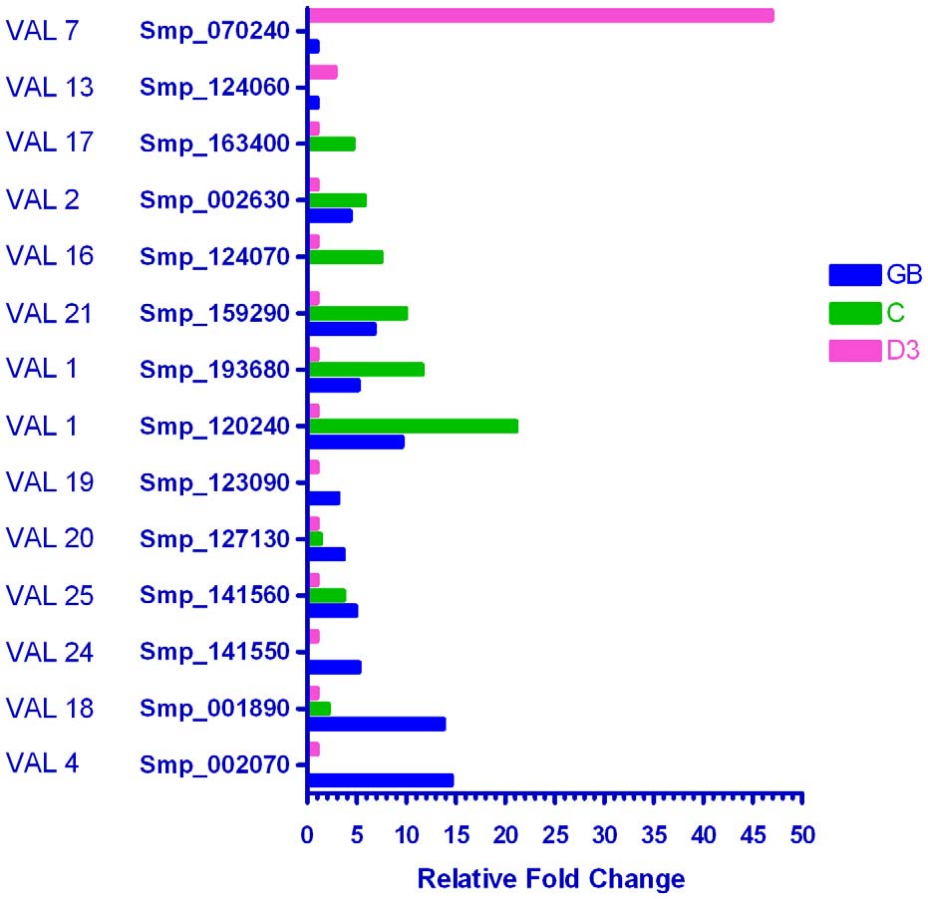
Genes encoding Venom Allergen-like proteins (VALs). Of the 28 VALs in the genome, 14 were shown to be differentially expressed in the three life cycle stages. Only two were up-regulated in the schistosomulum, six were most highly expressed in the cercaria, three of which were also up-regulated in the germ ball. Six were most highly expressed in the germ ball, two of which were also enriched in the cercaria. These expression patterns imply that the functions of these enigmatic proteins are mostly associated with entry into the mammalian host. Only the gene for VAL 7 appears to be activated after infection.

All proteins in this group possess a sperm coat protein (SCP) domain, and some, especially those derived from insects, act as allergens in the mammalian host, hence their name. Of the 28 schistosome VAL genes described by Chalmers *et al.* [32], 13 were differentially transcribed during the germ ball to day 3 schistosomulum transitions (46%; Figure 8). Most were enriched in the germ ball and/or cercaria compared to the day 3 schistosomulum, implying that their encoded proteins play a role in the early stages of infection. VALs 4, 24, 19, 20, 18, and 25 were most highly transcribed in the germ ball; of these, VALs 20 and 25 were also enriched in the cercaria. VALs 1, 21, 16, 2, and17 were most highly transcribed in the cercaria; of these, VALs 1, 21, and 2 were also up-regulated in the germ ball. (Note that VAL 1 is a faulty gene model represented by two overlapping Smps.) Only two VALs (7 and 13) were most highly transcribed in the schistosomulum, VAL 7 being ∼47 fold higher than the germ ball. This is noteworthy as none of the other VAL-encoding genes exhibited changes greater than 21 fold. Dillon *et al.* reported the up-regulation of VAL 7 (then Sm12775) in lung schistosomula [12], showing that its expression continues beyond the skin stage. Curwen *et al.* identified VALs 4, 10 and 18 in cercarial secretions by proteomics [2], while Chalmers *et al.* used qPCR to show that VALs 1, 4 and 10 were up-regulated in the cercaria [32], (the latter study was restricted to VALs 1–13). The one discrepancy with the previous reports is the absence of VAL 10 from our microarray analysis (transcripts were detected in the germ ball but did not reach the threshold of significance).

There are few clear indicators from the literature as to the function of VAL proteins. Hookworm Na-ASP-2 is the most abundant product released by *Necator americanus* L3 larvae upon skin penetration [82] and has been developed as a vaccine candidate [83]. It shows structural and charge similarity to host chemokines, suggesting that it may act as an extracellular ligand to an unknown receptor [84], while an *Onchocerca* VAL reportedly stimulates angiogenesis in the host [85]. By analogy, VAL 7 could feasibly interact with the host vasculature during parasite migration to the portal system.

#### Micro exon genes (MEGs)

The MEGs were briefly described by Berriman *et al.* [16] and a detailed account has been given by DeMarco *et al.* [33]. Greater than 80% of their protein coding regions comprise microexons ranging from six to 36 base pairs, the commonest frequency being 21. These genes are hypothesised to represent a mechanism by which schistosomes generate variant proteins potentially to confuse the immune system [33]. The MEG expression data has already been published in the comprehensive paper dealing with this unique collection of schistosome gene families [33]. MEGs show the highest fold changes encountered in this entire study, with eight having values greater than 40 fold (Figure S2). Of the 14 MEG families originally described [16], all but three were found to be highly transcribed in one of the stages under investigation. Only MEGs 1 and 11 showed no differential transcription during the transitions studied here, the former being consistently low and the latter undetectable. In contrast MEG 6, which was only two fold up-regulated in both cercaria and day 3 schistosomulum, was in fact highly transcribed at each stage. As many of the known MEGs exhibited very high fold changes of expression, other gene models of highly expressed transcripts annotated as ‘hypothetical protein’ or ‘expressed protein’ were manually inspected to see if they were MEGs. This led to the identification of four new MEGs that had no homology to previously identified MEG families, or to each other; they represent novel families, and were named MEGs 15–18, respectively [33]. MEGs 8 and 14 were highlighted as enriched in schistosomula in an earlier array experiment, before their unusual structure was identified (Sm12913/Smp_172180 and Sm01621/Smp_124000, respectively [12]).

MEGs 7, 10 and 18 were enriched in the germ ball and MEGs 4.2, 13 and 6 in the cercaria. MEGs 4.1, 8, 12, 14, 15, 16, and 17 were most highly transcribed in the day 3 schistosomulum and they were also up-regulated in the cercaria. MEGs 2, 3, 5, 8 and 9 were up-regulated solely in the day 3 schistosomulum. The greatly enhanced transcription of MEGs, in the day 3 schistosomulum, indicates the probable importance of their proteins for the parasite during establishment and subsequent persistence in the mammalian host, but the absence of any known domains, or of homology to proteins outside the genus *Schistosoma* makes interpretation of their function difficult.

#### Unannotated genes

A sizeable proportion of genes in the *S. mansoni* genome have no homology to genes encoding proteins of known function in other organisms, so cannot be classified on that basis. Nevertheless, significant numbers of their transcripts were differentially expressed. We report only the 58 transcripts with 10 fold or greater expression relative to at least one other stage (Tables S9, S10, S11). One of these, Smp_075420, is known to be up-regulated in day 2 and day 7 schistosomula [12]. Interrogation of the sequences with SignalP and HMMTOP2, for the presence of signal peptides and membrane spanning helices revealed 13 and 10, respectively. This suggests that a proportion of the products of the unannotated genes may be secreted or surface-exposed and thus capable of interacting with the external environment.

### Summary

In this paper we have described in detail the changes in gene transcription throughout the mammalian infection process. Evidence for DNA replication and cell division was only seen in the germ ball, while each stage up-regulated different genes involved in development and morphogenesis, including cell-cell adhesion. Thus, neural patterning genes were enriched in the germ ball, while there was considerable activation of genes involved in nerve function in the schistosomulum, coincident with body remodelling. Forward planning was seen throughout, with genes encoding skin penetration enzymes transcribed in the germ ball, and those involved in transformation, gut activity and tegument replacement beginning their up-regulation in the cercaria. Our data suggest there are vastly more proteases involved in skin penetration than hitherto envisaged. Likewise the number of possible gut proteases and transporters is expanded. The up-regulation of many receptors in the schistosomulum raises the thorny issue of whether they interact with host or endogenous ligands. Surprisingly, few stress-related genes were up-regulated in the schistosomulum and, apart from the cercarial enrichment of genes encoding proteins involved in aerobic respiration, there was little change in energy metabolism during the transitions studied. The inscrutable VALs are deployed early in infection, whilst the majority of the MEGS, also of unknown function, are transcribed later with extremely high fold changes. Many of the genes identified throughout our study warrant detailed investigation.

## Supporting Information

Figure S1 **Regression of microarray and qPCR data.** In order to validate the mircroarray data, the level of expression of selected genes was determined by real-time PCR with the same three biological replicates used for array hybridisation. To visualize the association between the two estimates, the mean and SEM log_2_ fold changes of the real time PCR values for six genes were plotted against the corresponding array data and a linear regression performed (r^2^ = 0.85; P<0.0001). In six instances the SEM was so small that it does not show on the graph. The two estimates of gene expression are in strong agreement.
(TIF)

Figure S2 **Microexon genes (MEGs).** The MEGs exhibited the highest fold changes in the entire study, ranging from 5–110× [33]. Twenty were up-regulated in the day 3 schistosomulum, with only four enriched in the germ ball and nine in the cercaria. The activation of this heterogeneous group of genes that encode secreted proteins of unknown function, is strongly associated with establishment in the mammalian host [33].
(TIF)

Table S1 **PCR Primers.** The selected genes used for qPCR validation of the array data are shown with their gene ID, annotation, and forward and reverse primers.
(DOC)

Table S2 **Anaerobic glycolysis.** In this and the subsequent tables the relative levels of transcription in the three life cycle stages are shown (germ ball, GB; cercaria, C; day 3 schistosomulum, D3), with the stage having the lowest expression set to one. Each locus is labeled with its systematic identity and annotation.
(DOC)

Table S3 **Stress-related genes.** Relative transcription levels of differentially transcribed genes related to stress responses.
(DOC)

Table S4 **Aspartic proteases.** Relative transcription levels of differentially transcribed genes encoding aspartic proteases.
(DOC)

Table S5 **Membrane: Transporters.** Relative transcription levels of differentially transcribed genes encoding membrane transporters.
(DOC)

Table S6 **Membrane: Channels.** Relative transcription levels of differentially transcribed genes encoding membrane channels.
(DOC)

Table S7 **Membrane: Receptors.** Relative transcription levels of differentially transcribed genes encoding receptor proteins.
(DOC)

Table S8 **Membrane: Structural proteins and enzymes.** Relative transcription levels of differentially transcribed genes encoding membrane structural proteins and enzymes.
(DOC)

Table S9 **Unannotated genes: Germ ball-enriched.** Relative transcription levels of unannotated genes in the germ ball compared to the other two stages.
(DOC)

Table S10 **Unannotated genes: Cercaria-enriched.** Relative transcription levels of unannotated genes in the cercaria compared to the other two stages.
(DOC)

Table S11 **Unannotated genes: Day 3 schistosomulum-enriched.** Relative transcription levels of unannotated genes in the day 3 schistosomulum compared to the other two stages.
(DOC)
