# Supplementary figures and images for: Gene Expression Patterns in Larval Schistosoma mansoni Associated with Infection of the Mammalian Host
Source: PLoS Negl Trop Dis. 2011 Aug 30;5(8):e1274. doi: 10.1371/journal.pntd.0001274 (PMC3166049; doi:10.1371/journal.pntd.0001274)

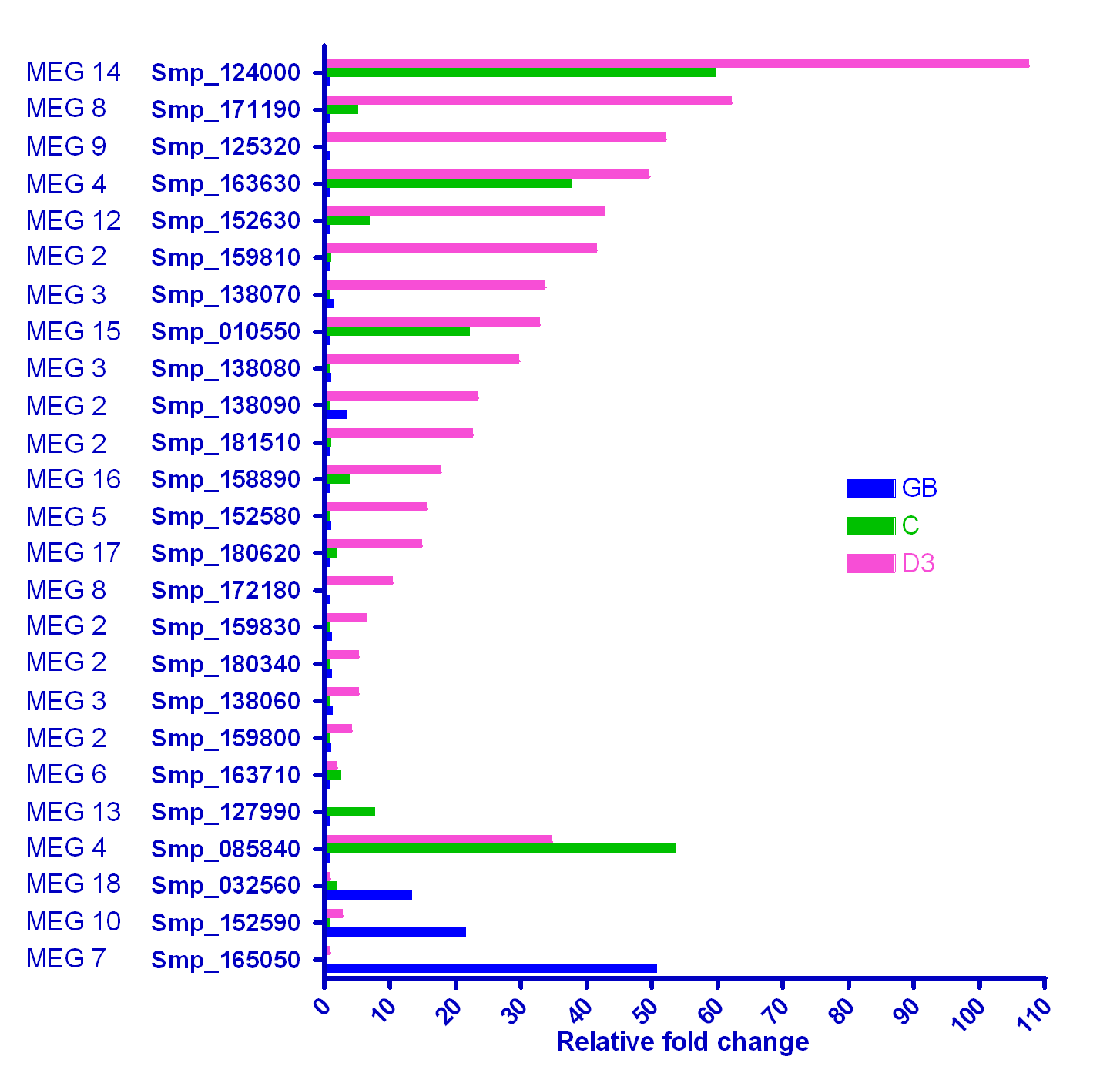

Supplement: Figure S2 — Microexon genes (MEGs). The MEGs exhibited the highest fold changes in the entire study, ranging from 5–110× [33]. Twenty were up-regulated in the day 3 schistosomulum, with only four enriched in the germ ball and nine in the cercaria. The activation of this heterogeneous group of genes that encode secreted proteins of unknown function, is strongly associated with establishment in the mammalian host [33]. (TIF) [file pntd.0001274.s002.tif]
