# Supplementary material for: Gene Expression Patterns in Larval Schistosoma mansoni Associated with Infection of the Mammalian Host
Source: PLoS Negl Trop Dis. 2011 Aug 30;5(8):e1274. doi: 10.1371/journal.pntd.0001274 (PMC3166049; doi:10.1371/journal.pntd.0001274)
Supplement: Table S1 — PCR Primers. The selected genes used for qPCR validation of the array data are shown with their gene ID, annotation, and forward and reverse primers. (DOC) [file pntd.0001274.s003.doc]

**Supporting** Table 1. PCR primers.

| **Gene ID** | **Product** | **Forward** | **Reverse** |
| --- | --- | --- | --- |
| Smp_079560 | MCM2 | CATGGTGGTGGGCTTTCAG | TGGCGGCGGATTCGT |
| Smp_159800 | MEG2 | CGGTAATGATTGTAAGGATGTCATAAA | CATTCCACGCTTTCTCAGGAA |
| Smp_124000 | MEG14 | CCTAAGAAACCACATGATGAACGT | ATCACTTCCCCAATAACTCCAAGT |
| Smp_082490 | Cyclin B | AAAATGCTGTCGTTGGCGTTA | CTAGGGCTGCGCGTGATC |
| Smp_179170 | Aspartic protease | TGAGAATGATGAACCTCCAATGA | TTTGACGATGTAGAGTATGCAATGG |
| Smp_165050 | MEG 7 | CTATTTTGATATGGCGAATGGTGTA | AGTAGCGGGTTTTGCTCTCACT |
| Smp_033040 | lactate dehydrogenase | CGAAAAGCCTGAACAAAAACTAGTAA | TGTGTCATTATATCAACTGGATTTGTG |
| Smp_044250 | metalloprotease | CGTCTGGATGCTCTCCTTAAGTC | TTCTGAGAACTGGACAGACAAACG |
|  | 18S | TCGGCGACGGATCTTTCA | CCGGAATCGAACCCTGATTC |
