# Supplementary material for: Gene Expression Patterns in Larval Schistosoma mansoni Associated with Infection of the Mammalian Host
Source: PLoS Negl Trop Dis. 2011 Aug 30;5(8):e1274. doi: 10.1371/journal.pntd.0001274 (PMC3166049; doi:10.1371/journal.pntd.0001274)
Supplement: Table S2 — Anaerobic glycolysis. In this and the subsequent tables the relative levels of transcription in the three life cycle stages are shown (germ ball, GB; cercaria, C; day 3 schistosomulum, D3), with the stage having the lowest expression set to one. Each locus is labeled with its systematic identity and annotation. (DOC) [file pntd.0001274.s004.doc]

Supporting Table 2 Anaerobic Glycolysis

| **Annotation** | **Gene ID** | **GB** | **C** | **D3** |
| --- | --- | --- | --- | --- |
| glucose-6-phosphate isomerase | Smp_022400 | - | 2.77 | 1.00 |
| 6-phosphofructokinase | Smp_043670.2 | - | 2.4 | 1.00 |
| malate dehydrogenase | Smp_047370 | - | 2.28 | 1.00 |
| hexokinase | Smp_043030 | 1.00 | - | 2.22 |
| 6-phosphofructokinase | Smp_043670.1 | 1.00 | - | 3.1 |
| L-lactate dehydrogenase | Smp_033040 | 1.00 | 1.49 | 14.7 |
