# Supplementary material for: Gene Expression Patterns in Larval Schistosoma mansoni Associated with Infection of the Mammalian Host
Source: PLoS Negl Trop Dis. 2011 Aug 30;5(8):e1274. doi: 10.1371/journal.pntd.0001274 (PMC3166049; doi:10.1371/journal.pntd.0001274)
Supplement: Table S3 — Stress-related genes. Relative transcription levels of differentially transcribed genes related to stress responses. (DOC) [file pntd.0001274.s005.doc]

Supporting Table 3 Stress-related genes

| **Annotation** | **Gene ID** | | **GB** | | **C** | | **D3** |
| --- | --- | --- | --- | --- | --- | --- | --- |
| Sm16 | Smp_113760 | | 14.12 | | 5.28 | | 1.00 |
| universal stress protein, putative | Smp_043120 | | 1.00 | | 2.15 | | - |
| glutathione peroxidase, putative | Smp_058690 | | 1.00 | | 2.15 | | - |
| multidrug resistance pump, putative | Smp_151290 | | - | | 3.01 | | 1.00 |
| Sm-p40 (heat shock protein) | Smp_049230 | | 1.56 | | 18.00 | | 1.00 |
| multidrug resistance pump, putative | Smp_135490 | | 1.00 | | - | | 4.05 |
| cu/zn superoxide dismutase, putative | Smp_174810 | | 1.00 | | - | | 5.75 |
| thioredoxin peroxidase, putative | Smp_059480 | 1.00 | | 5.97 | | 37.33 | |
