# Supplementary material for: Gene Expression Patterns in Larval Schistosoma mansoni Associated with Infection of the Mammalian Host
Source: PLoS Negl Trop Dis. 2011 Aug 30;5(8):e1274. doi: 10.1371/journal.pntd.0001274 (PMC3166049; doi:10.1371/journal.pntd.0001274)
Supplement: Table S4 — Aspartic proteases. Relative transcription levels of differentially transcribed genes encoding aspartic proteases. (DOC) [file pntd.0001274.s006.doc]

Supporting Table 4 Aspartic Proteases

| **Annotation** | **GeneID** | **GB** | **C** | **D3** |
| --- | --- | --- | --- | --- |
| subfamily A1A unassigned peptidase | Smp_136730 | 1 | - | 2.95 |
| subfamily A1A unassigned peptidase | Smp_018800 | 1 | - | 4.41 |
| subfamily A1A unassigned peptidase | Smp_136720 | 1 | - | 4.59 |
| subfamily A1A unassigned peptidase | Smp_136830.1 | 1 | 1.58 | 6.7 |
| subfamily A1A unassigned peptidase | Smp_136840 | 1 | - | 6.78 |
| subfamily A1A unassigned peptidase | Smp_136820 | 1 | - | 6.92 |
| subfamily A1A unassigned peptidase | Smp_132470 | 1 | 4.23 | 9.51 |
| subfamily A1A unassigned peptidase | Smp_132480 | 1 | 2.28 | 11.24 |
| subfamily A1A unassigned peptidase | Smp_136830.2 | 1 | 3.03 | 15.14 |
