# Supplementary material for: Gene Expression Patterns in Larval Schistosoma mansoni Associated with Infection of the Mammalian Host
Source: PLoS Negl Trop Dis. 2011 Aug 30;5(8):e1274. doi: 10.1371/journal.pntd.0001274 (PMC3166049; doi:10.1371/journal.pntd.0001274)
Supplement: Table S5 — Membrane: Transporters. Relative transcription levels of differentially transcribed genes encoding membrane transporters. (DOC) [file pntd.0001274.s007.doc]

Supporting Table 5 Membrane: Transporters

| **Annotation** | **Gene ID** | **GB** | **C** | **D3** |
| --- | --- | --- | --- | --- |
| Na-dependent neurotransmitter transporter | Smp_129900 | 1.00 | 14.12 | 7.20 |
| Na-dependent neurotransmitter transporter | Smp_129920 | - | 10.53 | 1.00 |
| Na-dependent neurotransmitter transporter | Smp_129910 | - | 5.91 | 1.00 |
| Na-bile acid cotransporter | Smp_131350 | 1.00 | 5.46 | 2.06 |
| monocarboxylate transporter | Smp_151010 | 1.00 | 4.56 | 2.13 |
| transporter | Smp_130890 | - | 2.66 | 1.00 |
| glycerol-3-phosphate transporter | Smp_084600.1 | - | 1.00 | 2.06 |
| mitochondrial glutamate carrier protein | Smp_044690 | - | 1.00 | 2.31 |
| smdr2 | Smp_055780 | - | 1.00 | 2.34 |
| solute carrier protein Zn | Smp_013440 | - | 1.00 | 2.61 |
| solute carrier family Zn | Smp_167630 | 1.00 | - | 2.63 |
| cation efflux protein/ zinc transporter | Smp_176100 | 1.00 | - | 2.70 |
| norepinephrine/norepinephrine transporter | Smp_157430 | 1.00 | - | 2.72 |
| high-affinity copper uptake protein | Smp_048230 | - | 1.00 | 2.90 |
| norepinephrine/norepinephrine transporter | Smp_157420 | 1.00 | -- | 3.07 |
| sugar transporter | Smp_129540 | 1.00 | 1.00 | 3.31 |
| multidrug resistance pump | Smp_135490 | 1.00 | 1.44 | 4.05 |
| stomatin-related | Smp_003440 | - | 1.00 | 4.20 |
| sodium/chloride dependent transporter | Smp_131890 | - | 1.00 | 4.45 |
| Monocarboxylate transporter | Smp_146830 | 1.22 | 1.00 | 6.00 |
| cationic amino acid transporter | Smp_123010 | 1.40 | 1.00 | 6.34 |
| glucose transport protein | Smp_105410 | 1.00 | - | 16.78 |
| cationic amino acid transporter | Smp_176930 | 1.00 | - | 19.43 |
