# Supplementary material for: Gene Expression Patterns in Larval Schistosoma mansoni Associated with Infection of the Mammalian Host
Source: PLoS Negl Trop Dis. 2011 Aug 30;5(8):e1274. doi: 10.1371/journal.pntd.0001274 (PMC3166049; doi:10.1371/journal.pntd.0001274)
Supplement: Table S6 — Membrane: Channels. Relative transcription levels of differentially transcribed genes encoding membrane channels. (DOC) [file pntd.0001274.s008.doc]

Supporting Table 6 Membrane: Channels

| **Annotation** | **Gene ID** | **GB** | **C** | **D3** |
| --- | --- | --- | --- | --- |
| amiloride-sensitive sodium channel-related | Smp_093210 | - | 7.95 | 1.00 |
| expressed protein voltage calcium channel | Smp_165660 | - | 4.00 | 1.00 |
| twik family of potassium channels | Smp_034850 | - | 3.70 | 1.00 |
| twik family of potassium channels | Smp_141570 | - | 3.25 | 1.00 |
| anion exchange protein | Smp_180950 | - | 2.21 | 1.00 |
| voltage-gated potassium channel | Smp_161140 | - | 1.92 | 1.00 |
| voltage-gated potassium channel | Smp_194710 | 1.00 | - | 2.28 |
| anion exchange protein | Smp_136030 | - | 1.00 | 2.32 |
| mitochondrial import receptor subunit tom40 | Smp_042560 | - | 1.00 | 2.35 |
| voltage-gated potassium channel | Smp_160780 | 1.59 | 1.00 | 4.00 |
| voltage-gated potassium channel | Smp_094560 | 1.00 | 0.94 | 4.06 |
| voltage-gated potassium channel | Smp_121190 | 1.82 | 1.00 | 4.33 |
| aquaporin-3 | Smp_005740 | 1.00 | - | 19.39 |
