# Supplementary material for: Gene Expression Patterns in Larval Schistosoma mansoni Associated with Infection of the Mammalian Host
Source: PLoS Negl Trop Dis. 2011 Aug 30;5(8):e1274. doi: 10.1371/journal.pntd.0001274 (PMC3166049; doi:10.1371/journal.pntd.0001274)
Supplement: Table S7 — Membrane: Receptors. Relative transcription levels of differentially transcribed genes encoding receptor proteins. (DOC) [file pntd.0001274.s009.doc]

Supporting Table 7 Membrane: Receptors

| **Annotation** | **Gene ID** | **GB** | **C** | **D3** |
| --- | --- | --- | --- | --- |
| opsin-like receptor | Smp_164140 | 9.85 | 1.12 | 1.00 |
| muscarinic acetylcholine (GAR) receptor | Smp_145540 | - | 2.88 | 1.00 |
| GPCR | Smp_099670 | - | 5.36 | 1.00 |
| progestin and adipoq receptor family member VI | Smp_086190 | 1.00 | - | 2.00 |
| rhodopsin-like orphan GPCR | Smp_072450 | - | 1.00 | 2.09 |
| G-protein coupled receptor fragment | Smp_152540 | 1.00 | 1.24 | 2.30 |
| G-protein coupled receptor fragment | Smp_132220 | 1.03 | 1.00 | 2.34 |
| biogenic amine (dopamine) receptor | Smp_127310 | 1.00 | - | 2.43 |
| P2X receptor subunit (AJ703803) | Smp_179310 | 1.00 | - | 2.81 |
| adiponectin receptor | Smp_045410 | - | 1.00 | 2.83 |
| glutamate receptor, kainate | Smp_153780 | - | 1.00 | 4.88 |
| CD36-like class B scavenger receptor | Smp_011680 | - | 1.00 | 8.91 |
