# Supplementary material for: Gene Expression Patterns in Larval Schistosoma mansoni Associated with Infection of the Mammalian Host
Source: PLoS Negl Trop Dis. 2011 Aug 30;5(8):e1274. doi: 10.1371/journal.pntd.0001274 (PMC3166049; doi:10.1371/journal.pntd.0001274)
Supplement: Table S8 — Membrane: Structural proteins and enzymes. Relative transcription levels of differentially transcribed genes encoding membrane structural proteins and enzymes. (DOC) [file pntd.0001274.s010.doc]

Supporting Table 8 Membrane: structure and enzyme

| **Annotation** | **Gene ID** | **GB** | **C** | **D3** |
| --- | --- | --- | --- | --- |
| Ca-transporting ATPase SR/ER type | Smp_136710 | - | 12.45 | 1.00 |
| atpase, class VI, type 11c (Flippase) | Smp_175790 | 1.00 | 4.11 | 2.30 |
| Tetraspanin-18 (Tspan-18) | Smp_174190 | - | 1.00 | 2.43 |
| innexin | Smp_129020 | 1.00 | - | 3.08 |
| na+/k+ atpase alpha subunit | Smp_015020 | 1.00 | - | 3.41 |
| fer-1-related | Smp_141010 | - | 1.00 | 3.48 |
| hypothetical protein | Smp_191900 | 1.00 | - | 2.54 |
| peptidyl-glycine alpha-amidating monooxygenase | Smp_156530 | 1.08 | 1.00 | 2.71 |
| innexin | Smp_026570 | 1.00 | - | 3.53 |
| expressed protein | Smp_140140 | 1.00 | - | 3.83 |
| integrin alpha | Smp_126140 | - | 1.00 | 4.32 |
| tetraspanin | Smp_131840 | - | 1.00 | 5.17 |
| innexin | Smp_141290 | - | 1.00 | 5.35 |
| tetraspanin | Smp_099770 | 1.00 | - | 9.38 |
| tetraspanin D76 | Smp_041460 | 1.00 | - | 15.89 |
| similar to tetraspanin TE736 | Smp_194980 | 1.00 | 1.20 | 21.56 |
| tetraspanin | Smp_059530 | 1.00 | - | 67.23 |
