# Supplementary material for: Gene Expression Patterns in Larval Schistosoma mansoni Associated with Infection of the Mammalian Host
Source: PLoS Negl Trop Dis. 2011 Aug 30;5(8):e1274. doi: 10.1371/journal.pntd.0001274 (PMC3166049; doi:10.1371/journal.pntd.0001274)
Supplement: Table S9 — Unannotated genes: Germ ball-enriched. Relative transcription levels of unannotated genes in the germ ball compared to the other two stages. (DOC) [file pntd.0001274.s011.doc]

Supporting Table 9 Unnannotated genes: Germ ball-enriched

| **Gene ID** | **Fold Change vs C^a^** | **Fold Change vs D3^b^** | **SignalP**  **Y/N^c^** | **HMMTOP2-predicted**  **transmembrane helices^d^** |
| --- | --- | --- | --- | --- |
| Smp_178960 | - | 27.84 | N | 0 |
| Smp_102670 | - | 23.72 | N | 0 |
| Smp_019000 | - | 23.1 | Y | 2 |
| Smp_193370 | 14.03 | 17.61 | N | 0 |
| Smp_032560 | - | 13.43 | Y | 0 |
| Smp_035550 | - | 12.51 | N | 0 |
| Smp_166690 | - | 12.22 | N | 0 |
| Smp_113710 | - | 11.3 | N | 0 |
| Smp_098020 | 10.81 | 11.27 | Y | 0 |
| Smp_161820 | - | 11.25 | N | 0 |
| Smp_143460 | 10.53 | 11.13 | N | 0 |
| Smp_138560 | - | 10.68 | N | 3 |
| Smp_100560 | - | 10.59 | N | 0 |
| Smp_177540 | 22.23 | - | N | 0 |
| Smp_065210 | 20.43 | - | N | 0 |
| Smp_132950 | 14.63 | - | N | 0 |
| Smp_119910 | 14.13 | - | N | 0 |
| Smp_072560 | 12.58 | - | N | 0 |
| Smp_013970 | 12.36 | - | N | 0 |
| Smp_193330 | 11.76 | - | N | 0 |
| Smp_164950 | 10.67 | - | N | 0 |

^a^ Relative fold change in the germ ball compared to the cercaria (set to 1)

^b^ Relative fold change in the germ ball compared to the day 3 schistosomulum (set to 1)

^c^ Presence (Y) or absence (N) of a signal peptide as predicted by SignalP

^d^ Number of transmembrane helices predicted by HMMTOP2
