# Supplementary material for: Gene Expression Patterns in Larval Schistosoma mansoni Associated with Infection of the Mammalian Host
Source: PLoS Negl Trop Dis. 2011 Aug 30;5(8):e1274. doi: 10.1371/journal.pntd.0001274 (PMC3166049; doi:10.1371/journal.pntd.0001274)
Supplement: Table S10 — Unannotated genes: Cercaria-enriched. Relative transcription levels of unannotated genes in the cercaria compared to the other two stages. (DOC) [file pntd.0001274.s012.doc]

Supporting Table 10 Unnanotated genes: cercaria-enriched

| **Gene ID** | **Fold Change vs GB^a^** | **Fold Change vs D3^b^** | **SignalP**  **Y/N^c^** | **HMMTOP2-predicted**  **Transmembrane helices^d^** |
| --- | --- | --- | --- | --- |
| Smp_088090 | 22.79 | 15.75 | N | 0 |
| Smp_133830 | 21.2 | - | N | 0 |
| Smp_194420 | 16.38 | - | N | 0 |
| Smp_074450 | 14.38 | - | N | 0 |
| Smp_057860 | 14.17 | - | Y | 0 |
| Smp_181690 | 14 | - | N | 0 |
| Smp_177640 | 13.24 | - | N | 0 |
| Smp_172450 | 12.55 | - | N | 0 |
| Smp_172460 | 12.43 | - | N | 0 |
| Smp_071050 | 12.04 | - | Y | 0 |
| Smp_022450 | 11.73 | - | Y | 3 |
| Smp_156510 | 11.72 | - | N | 0 |
| Smp_161030 | 11.66 | - | N | 0 |
| Smp_105610 | 11.44 | 11.17 | N | 0 |
| Smp_074560 | 11.38 | 10.77 | N | 0 |
| Smp_035290 | 11.2 | 11.56 | N | 0 |
| Smp_129090 | 10.64 | - | N | 0 |
| Smp_075420 | 10.63 | - | N | 0 |
| Smp_125140 | 10 | - | N | 1 |
| Smp_102670 | - | 38.88 | N | 0 |
| Smp_018880 | - | 36.79 | N | 1 |
| Smp_156760 | - | 33.97 | N | 0 |
| Smp_178960 | - | 20.16 | N | 0 |
| Smp_189240 | - | 14.66 | N | 0 |
| Smp_076010 | - | 11.96 | N | 0 |
| Smp_193850 | - | 11.47 | N | 2 |
| Smp_194040 | - | 10.81 | Y | 0 |
| Smp_029930 | - | 10.45 | N | 0 |

^a^ Relative fold change in the cercaria compared to the germ ball (set to 1)

^b^ Relative fold change in the cercaria compared to the day 3 schistosomulum (set to 1)

^c^ Presence (Y) or absence (N) of a signal peptide as predicted by SignalP

^d^ Number of transmembrane helices predicted by HMMTOP2
