# Supplementary material for: Gene Expression Patterns in Larval Schistosoma mansoni Associated with Infection of the Mammalian Host
Source: PLoS Negl Trop Dis. 2011 Aug 30;5(8):e1274. doi: 10.1371/journal.pntd.0001274 (PMC3166049; doi:10.1371/journal.pntd.0001274)
Supplement: Table S11 — Unannotated genes: Day 3 schistosomulum-enriched. Relative transcription levels of unannotated genes in the day 3 schistosomulum compared to the other two stages. (DOC) [file pntd.0001274.s013.doc]

Supporting Table 11. Unnannotated genes: Day 3 schistosomulum-enriched

| **Gene ID** | **Fold Change vs GB^a^** | **Fold Change vs C^b^** | **SignalP**  **Y/N^c^** | **HMMTOP2-predicted**  **transmembrane helices^d^** |
| --- | --- | --- | --- | --- |
| Smp_194860 | 39.79 | 20.64 | Y | 0 |
| Smp_075420 | 25.34 | - | N | 0 |
| Smp_114660 | 23.04 | - | N | 1 |
| Smp_074450 | 22.61 | - | N | 0 |
| Smp_016490 | 18.34 | - | Y | 0 |
| Smp_081900 | 16.26 | - | N | 1 |
| Smp_129090 | 16.11 | - | N | 0 |
| Smp_163520 | 14.51 | - | N | 0 |
| Smp_081920 | 13.58 | 13.78 | Y | 1 |
| Smp_167280 | 12.55 | - | N | 0 |
| Smp_071050 | 11.94 | - | Y | 0 |
| Smp_064300 | 11.91 | 15.43 | Y | 0 |
| Smp_141500 | 11.83 | - | Y | 0 |
| Smp_075500 | - | 19.9 | Y | 3 |
| Smp_065370 | - | 11.52 | N | 0 |

^a^ Relative fold change in the day 3 schistosomulum compared to the germ ball (set to 1)

^b^ Relative fold change in the day 3 schistosomulum compared to the cercaria (set to 1)

^c^ Presence (Y) or absence (N) of a signal peptide as predicted by SignalP

^d^ Number of transmembrane helices predicted by HMMTOP2
